# Diamondoids and thiadiamondoids generated from hydrothermal pyrolysis of crude oil and TSR experiments

**DOI:** 10.1038/s41598-021-04270-z

**Published:** 2022-01-07

**Authors:** Yanyan Peng, Chunfang Cai, Chenchen Fang, Liangliang Wu, Jinzhong Liu, Peng Sun, Dawei Liu

**Affiliations:** 1 Key Laboratory of Cenozoic Geology and Environment, Institute of Geology and Geophysics, Chinese Academy of Sciences, Beijing, 100029 People’s Republic of China; 2 Innovation Academy for Earth Science, Chinese Academy of Sciences, Beijing, 100029 People’s Republic of China; 3 College of Earth and Planetary Sciences, University of Chinese Academy of Sciences, Beijing, 100049 People’s Republic of China; 4 Key Laboratory of Exploration Technologies for Oil and Gas Resources of Ministry of Education, Yangtze University, Wuhan, 430100 Hubei People’s Republic of China; 5 State Key Laboratory of Organic Geochemistry (SKLOG), Guangzhou Institute of Geochemistry, Chinese Academy of Sciences, Guangzhou, 510640 People’s Republic of China; 6 PetroChina Research Institute of Petroleum Exploration and Development, Beijing, 100083 People’s Republic of China

**Keywords:** Biogeochemistry, Energy science and technology

## Abstract

Diamondoid compounds are widely used to reflect thermal maturation of high mature source rocks or oils and oil cracking extents. However, diamondoids and thiadiamondoids were demonstrated to have newly been generated and decomposed in our hydrothermal pyrolysis of crude oil and TSR experiments. Our results show that adamantanes and diamantanes are generated primarily within the maturity range 0.48–2.1% and 1.2–3.0% EasyRo, respectively. Their formation is enhanced and the decomposition of diamantanes obviously lags at elevated temperatures compared with anhydrous experiments. MDI, EAI, DMAI-1, DMDI-2 may serve as reliable maturity proxies at > ca.1.0% EasyRo, and other isomerization indices (TMAI-1, TMAI-2 and DMAI-2) are effective for the highly mature organic matter at EasyRo > 2.0%. The extent of oil cracking (EOC) calculated from the broadly used (3- + 4-) MD method (Dahl et al. in Nature 399:54–56, 1999) is proven to overestimate, especially for highly cracked samples due to the new generation of (3- + 4-) MD. Still, it can be corrected using a new formula at < 3.0% EasyRo. Other diamondoid-related indices (e.g., EAI, DMDI-2, As/Ds, MAs/MDs, DMAs/DMDs, and DMAs/MDs) can also be used to estimate EOC. However, these indices cannot be applied to TSR-altered petroleum. TSR is experimentally confirmed to generate diamantanes and thiaadmantanes at 1.81% EasyRo likely via direct reactions of reduced S species with hydrocarbons and accelerate the decomposition of diamantanes at > 2.62% EasyRo compared with thermal chemical alteration (TCA). More studies are needed to assess specific mechanisms for the formation of thiadiamondoids under natural conditions.

## Introduction

Nanometer-sized polycyclic diamondoid hydrocarbons (also polymantanes) appear in petroleum (crude oil and condensates), coal and sedimentary rock in the geosphere^1–8^, and are considered to form during early diagenesis^7,9,10^. Results of numerous laboratory syntheses suggest that diamondoids can generate in mudstone and shale source rocks by carbonium ion rearrangements of specific strained polycyclic alkane precursors under thermal stress in the presence of strong Lewis acids acting as catalysts^4,11,12^. Diamondoids have high thermal stability because they possess a unique ring system composed of cages with three or more fused chair cyclohexane rings. They are considered stable when other hydrocarbons are being cracked down. Therefore, diamondoids provide a measure of the degree of thermal maturation using their isomerization proxies, and (3- + 4-) methyl diamondoid (3- + 4-MD) concentrations can be used to reflect oil cracking extents^5^.

However, diamondoids have been formed by the pyrolysis of crude oils^13^ and all four oil fractions^14–16^, as well as compounds, such as C_16_, C_19_, C_22_, C_34_ and C_36_ n-alkanes^17^ and β-ionone^18^ without catalysis. All these pyrolysis experiments were conducted in dry conditions, ignoring the effect of water on oil cracking. It is typically recognized that as a ubiquitous substance in sedimentary basins, water can react with organic compounds to provide hydrogen atoms and may have been involved in quite many reactions^19–22^, hydrothermal pyrolysis of organic matter at elevated temperatures have been shown to generate gases more similar to natural gases^23,24^. On the other hand, diamondoid has been proposed to create by thermochemical sulfate reduction^25^ (TSR), a process whereby aqueous sulfate and petroleum compounds react at high temperatures (≥ 120 °C) to result in elevated H_2_S concentrations in many carbonate reservoirs^26–29^. This is based on the evidence that TSR-altered oils and condensates in the Cambrian and Ordovician in the Tarim basin and Smackover Formation in the US Gulf Coast have much higher concentrations of diamondoids than non- or minor TSR-altered oils which experienced higher heating.

The diamondoid isomerization ratios are used to assess the thermal maturity of crude oils and source rocks^30–32^ based on the more stable thermodynamic properties of bridge carbon substitution in isomers^4,10,33^. There are nine isomerization indices: MAI [1-MA/(1-MA + 2-MA)]; EAI [1-EA/(1-EA + 2-EA)]; DMAI-1 [1,3-DMA/(TMA + 1,3,4-TMA)]; TMAI-2 [1,3,5-TMAI/(1,3,5-TMA + 1,3,6-TMA)]; MDI [4-MD/(4-MD + 1-MD + 3-MD)]; DMDI-1 [4,9-DMD/(4,9-DMD + 3,4-DMD)]; and DMDI-2 [4,9-DMD/(4,9-DMD + 4,8-DMD)]^9,10,13,16,30,32,34^. However, the maturity scopes for the application of each index are still controversial. Also, the isomerization of diamondoids is proposed to enhance due to TSR. As a result, diamondoid-based proxies cannot be used to reflect maturity and lithology in the TSR active areas^25^. However, these proposals have not been confirmed from simulation experiments and the mechanisms for the generation of diamondoids from TSR remain confused.

Thiadiamondoids are diamond-like compounds with a sulfide bond located within the cage structure. Hanin et al.^35^ found that alkylated 2-thiaadamantanes were present only in TSR-altered oils and thus proposed that alkylated 2-thiaadamantanes might have been formed by acid-catalyzed rearrangement of tricyclic sulfide. Wei et al.^36^ showed a linear correlation between the concentrations of thiadiamondoids and diamondoids in support of their diamondoids origin. Some laboratory experiments were carried out to address the origins of the thiadiamondoids. There may be at least two distinct mechanisms for the formation of thiadiamondoids, one under relatively low temperatures and the other at high temperatures. Wei et al.^37^ found that trace amounts of dimethyl-2-thiaadamantanes were produced by montmorillonite K10-catalyzed rearrangement of thiocholesterol at 200 °C. Such an origin of dimethyl-2-thiaadamantanes may have occurred in extractable organic matter or been bound in kerogen of source rocks during early diagenesis. Thiadiamondoids are also shown to form from reactions between diamondoids or diamondoidthiols and sulfate or sulfur species at ≥ 350°C^37,38^. However, no detectable thiadiamondoids were generated from the experiments of a diamondoid-enriched condensate with CaSO_4_ or S^0^ at 360 °C for 20 h or 40h^38^, suggesting that no thiadiamondoids have been generated under a lab condition similar to natural geologic environments.

In the present study, hydrothermal pyrolysis and TSR experiments were carried out under the same experimental conditions as those of anhydrous pyrolysis of Fang et al.^13^. The objectives of this study are to: (1) clarify the effect of water on yields of diamondoids; (2) ascertain whether TSR will lead to the new generation of diamondoids and thiadiamondoids; (3) calibrate the reliable EasyRo maturity range of isomerization-related diamondoid proxies; (4) develop diamondoid-related indices to reflect oil cracking extents (EOCs). This study will have a broad application in petroleum evaluation and thus exploration.

## Experimental methods and samples

### Sample preparation

A typical black oil (53.1% saturated, 16.0% aromatic, 15.4% resin, 3.86% asphaltene, and 12% other components) was collected from the HD23 well of the Tarim Basin, NW China, used by Fang et al.^13,16^. The oil contains well-preserved, mono-modal distribution of n-alkanes, and abundant biomarkers and has not undergone obvious biodegradation and thermal degradation. This oil is in the early stage of the oil generation window (Ro of 0.6%–0.8%) as indicated by some maturity proxies, such as the methylphenanthrene index (MPI) = 0.62, %Rc (= 0.60 * MPI-1 + 0.37) = 0.74; C29 steranes ββ/(ββ + αα) = 0.67, C29 steranes 20S/(20S + 20R) = 0.47, and C31 hopanes 22S/(22S + 22R) = 0.53. More details can be found in Fang et al.^16^. Quantitative analysis showed that this oil contained relatively low concentrations of adamantanes and diamantanes (359 μg/g for adamantanes and 79.8 μg/g for diamantanes). Therefore, this oil is suitable to study the evolution of diamondoids during thermal maturation.

Another oil sample (ZS1-L oil) was obtained from the ZS1 well in the Tazhong of the Tarim basin. This oil has a low sulfur content of 0.18%, API gravity of 48.3°, viscosity of 1.60 mPa·s, density of 0.789 g/cm^3^ at 20 °C and is composed of saturates (84.2%), aromatics (5.5%), resins (4.6%) and asphaltene (5.8%). The diamondoids and thiaadmantanes concentrations of ZS1-L oil are about 1861 μg/g (1697 μg/g for As, 127 μg/g for Ds and 37 μg/g for (3- + 4-) MD), 19 μg/g, respectively. ZS1-L oil produced from the Cambrian, which experienced higher heating, show much less thiadiamondoid (< 20 ug/g), less DBT/Phen ratios (< 2.0) and they have δ^34^S value of + 23.3‰and most ^13^C depleted n-alkanes, implying the lowest (negligible) degree of TSR alteration^25^.

To eliminate the effect of original diamondoids on the quantification of the diamondoid generated during oil cracking, both HD23 oil and ZS1-L oil were evaporated in a fume hood for 120 h before the pyrolysis experiment to remove the original adamantanes according to the method described by Fang et al.^13^. GC–MS–MS and GC–MS showed that no adamantanes and thiaadmantanes have remained in the evaporated oil. That means both thiaadmantanes and admantanes had been volatilized before experiments. Inorganic reagents, including MgSO_4_ with δ^34^S of + 3.75‰, elemental S with δ^34^S of − 6.3‰, CaSO_4_·2H_2_O with δ^34^S of + 21.3‰, sodium chloride (NaCl) and magnesium chloride (MgCl_2_), were purchased from Sigma–Aldrich (St. Louis, MO) and are analytical grade (> 99.9% purity).

### Confined pyrolysis experiments

Pyrolysis experiments were conducted using two methods, gold tubes and quartz tubes, depending on the volumes of tubes. For TSR experiments, the liquid chromatographic (LC) separation of thiadiamondoids needs to recover sufficient pyrolysates (pyrolysis products). Hence, gold tubes were used for hydrothermal experiments and quartz tubes were used for TSR experiments. The thermal maturation of samples was calculated using the Easy%Ro approach developed by Sweeney and Burnham^39^. The pyrolysates were collected and analyzed using GC–MS and GC–MS–MS.

#### Quartz tube pyrolysis experiments

110 mm-long quartz tubes with 20 mm internal diameter, 1 mm thick wall, giving a total reactor volume of approximately 25 mL, were used for the TSR Experiments. Before loading the tubes, each tube was cleaned using distilled milli-Q water and heated to 450 °C. The solid or liquid reactants were accurately loaded or injected into tubes by a small funnel with an outside diameter slightly smaller than the inner diameter of the quartz tubes. After that, the other end of the tubes was sealed under vacuum conditions. Finally, the tubes with samples loaded were put into autoclaves and desired temperature programs were carried out. After the desired temperature or time was reached, each autoclave was quenched to room temperature before being opened.

We used the Mg^2+^-talc-silica system as a mineral buffer at elevated temperatures to keep the in-situ pH in a narrow range (pH ~ 3)^40^. Thus, each quartz tube was loaded with 30 mg talc, 30 mg silica, 100 mL distilled milli-Q water solution with 5.6 wt.% MgCl_2_, 10 wt.% NaCl and 0.56 wt.% MgSO_4_. The approach used to regulate in situ chemical conditions for our study relies on chemical reactions known to proceed rapidly at the temperature and pressure conditions of the experiments^41,42^. More details related to the mineral buffer approach are given by Zhang et al.^40,43^. Subsequently, 100 mg of ZS1-L oil sample, 25 mg elemental S and 100 mg MgSO_4_ were accurately weighed and transferred to the tubes by a small funnel with an outside diameter slightly smaller than the inner diameter of the quartz tubes (Group 1). Blank experiments (TCA experiments) with 100 mg of ZS1-L oil sample and 100 mL solution (5.6 wt.% MgCl_2_, 10 wt.% NaCl) were performed in parallel. After being sealed under vacuum conditions, the quartz tubes were placed in stainless steel autoclaves and heated from 336 °C to 600 °C at constant heating rates (20 °C /h). The error of the recorded temperatures is <  ± 1 °C. When the desired temperature or time was reached, each autoclave was quenched to room temperature before being opened.

After pyrolysis, the tubes were placed in a vacuum glass system connected to the GC inlet and then pulled out. After cracking the quartz tube, gaseous hydrocarbons were released and introduced into the GC system. Part of the gas collected at each temperature was bubbled through a basic 5% AgNO_3_ solution to convert H_2_S to Ag_2_S for isotopic analysis, as discussed in Sect. 2.4. The individual gaseous hydrocarbons were quantified using an Agilent Technologies 6890 N gas chromatograph and the pyrolysates (pyrolysis products) were recovered by repeated sonication with dichloromethane. The organic fraction then LC separated to saturate, aromatic and sulfidic fractions using silver nitrate impregnated silica gel column as described below.

#### Gold tube pyrolysis experiments

Oil pyrolysis in hydrothermal conditions was conducted in sealed gold tubes with an internal diameter of 5 mm and wall thickness of 0.5 mm after the method of Fang et al.^13^. Each tube was between 40 and 50 mm long, giving a total reactor volume of approximately 0.5 mL. One end of each tube was crimped and sealed using an argon arc welder. Before loading the samples, the open-ended tubes were heated to 600 ℃ to remove any residual organic material. Then, specific amounts of samples (i.e., oil and water with the weight ratio of 1:1) were loaded into the gold tubes, which were subsequently flushed with argon for 5 min and sealed under an argon atmosphere. Individual sealed gold tubes were later placed in separate stainless-steel autoclaves and inserted into a pyrolysis oven. The ovens were heated from 336 to 600 ℃ at two constant rates of 20 °C/h and 2 °C/h, respectively, under the constant pressure of 50 MPa. After reaching desired reaction temperature and the pressure was released, the tubes were taken out from autoclaves.

Two parallel gold tubes were positioned in each autoclave to quantify diamondoid hydrocarbons and the extent of oil cracking (EOC) in pyrolysates. To remove any potential organic contaminants from the exterior of the gold tubes, they were cleaned in dichloromethane and allowed to air dry. Tubes were cooled for 25–30 min using liquid nitrogen following this cleaning procedure. Upon removing the liquid nitrogen, the first cleaned gold tube for diamondoid analysis was rapidly cut in half and placed in a 4 ml sample vial filled with isooctane to minimize loss of volatile components. The parallel gold tube for EOC analysis was first cut off welded ends and then rapidly cut into four equal pieces. The four tube pieces were quickly placed into a 10 ml sample vial filled with dichloromethane and allowed to soak overnight (12–20 h). The vials containing the gold tube pieces were then sonicated repeatedly to recover the pyrolysates. The vials were then opened for a minimal amount of time to remove the pieces of gold tubing and their transfer to 4 mL sample vial containing dichloromethane. Asphaltenes were then precipitated from the products by adding 50-fold (volume ratio for n-hexane/bitumen) cold n-hexane and removed by centrifugation. Then the absolute amount of liquid hydrocarbon was weighed on residual liquid hydrocarbons.

### TSR control-experiments

Although anhydrite appears to be the reactive oxidant and is replaced by calcite and dolomite in natural TSR reservoirs^26–28,44–48^, it is generally not used in laboratory TSR studies due to its low solubility^17,49–51^. Magnesium (Mg^2+^) is always present in natural TSR reservoirs and may play a catalytic role in natural TSR processes. To ensure that the sulfate will be involved in TSR experiments, rather than just elemental S, another group of TSR control-experiments using elemental S and CaSO_4_·2H_2_O was conducted (Group 2). Therefore, there are two sulfates with large different sulfur isotope values (MgSO_4_: 3.74‰; CaSO_4_·2H_2_O: 21.3‰) were used in the present study for the comparison of the δ^34^S values of H_2_S. Group1: See 2.2.1 for details; Group2: 100 mg of ZS1-L oil sample, 25 mg elemental S, 100 mg CaSO_4_·2H_2_O and 100 mL solution (5.6 wt.% MgCl_2_, 10 wt.% NaCl and 0.8 wt.% CaSO_4_·2H_2_O). In Group 2 experiments, the pyrolysis temperature and time were 360 ºC and 48–840 h, respectively. The experimental conditions are consistent with Group 1.

### Quantification of diamondoids (GC–MS-MS) and thiaadamantanes (GC–MS)

About 50 µL standards isooctane with n-dodecane-d_26_ and n-hexadecane-d_34_ were injected into the sample vial. The vial was ultrasonically treated for 10 min to improve the dissolution of pyrolysates. Leaving the vial for 12 h to precipitate asphaltenes, a volume of the supernatant was transferred into a 2 ml auto-sampler vial for GC–MS-MS. The identification and measurement of diamondoids using the GC–MS–MS method was described in detail elsewhere^52^.

The liquid chromatographic (LC) separation of thiadiamondoids was done according to the method of Wei et al.^36^: LC on silver nitrate-impregnated silica gel was used to fractionate samples into saturate, aromatic, and sulfidic fractions by sequential elution using hexane, dichloromethane, and acetone, respectively. Care was taken to avoid drying the sulfidic fractions during evaporation and concentration to smaller volumes down to 50–150 μl and analyzed for thiaadamantanes using GC–MS method as detailed in Cai et al.^25^.

### Sulfur isotope analysis

For analysis of δ^34^S of the H_2_S (converted to Ag_2_S) was conducted at the Institute of Geology and Geophysics, Chinese Academy of Sciences. The dried Ag_2_S and Cu_2_O were generally mixed in a proportion of 1:10 and then combusted at 1100 °C under vacuum to produce SO_2_. The resulting SO_2_ was sealed within pyrex tubing and analyzed on a Thermo Delta S mass spectrometer. Sulfur isotope values are expressed as per mil (‰) deviations from the sulfur isotope composition of the Vienna Canyon Diablo Troilite (VCDT) using the conventional delta (δ^34^S) notation. Isotopic results were generally reproducible within ± 0.3‰.

## Results

The yield of the individual diamondoid compounds is used to characterize the variation in the absolute amount of diamondoids during the experiments and expresses as the mass of diamondoids generated at each temperature point relative to the initial weight of the oil in each gold tube or quartz tube, according to

1 $$Y_{i} = M_{i} {/}M_{0}$$

where *Y*_*i*_ is the yield of the particular diamondoids (e.g., an individual diamondoid compound, a group of compounds, or the total diamondoids); *M*_*i*_ is the mass (µg) of the relevant diamondoids; *M*_0_ is the initial mass (g) of the diamondoid-generating substance (original oil mass loaded in the tube).

**Figure 1:**
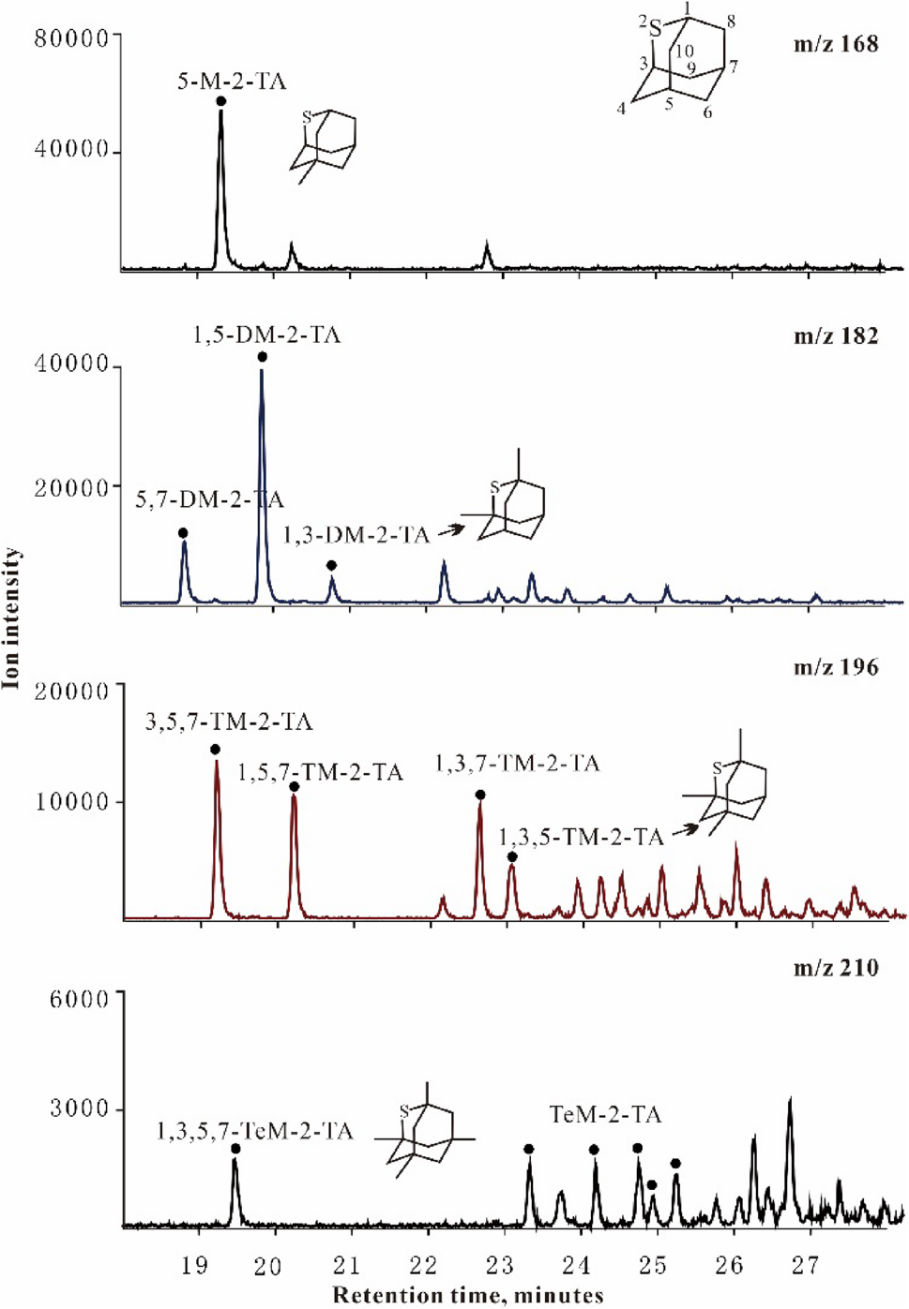
Mass chromatograms of alkylated thiadiamantanes in the sulfidic fraction of the products from TSR experiments (Group1) at 480 °C. 5-M-2-TA = 5-methyl-2-thiaadmantane; 5,7-DM-2-TA = 5,7-dimethyl-2-thiaadmantane; 1,5-DM-2-TA = 1,5-dimethyl-2-thiaadmantane; 1,3-DM-2-TA = 1,3-dimethyl-2-thiaadmantane; 3,5,7-TM-2-TA = 3,5,7-trimethyl-2-thiaadmantane; 1,5,7-TM-2-TA = 1,5,7-trimethyl-2-thiaadmantane; 1,3,7-TM-2-TA = 1,3,7-trimethyl-2-thiaadmantane; 1,3,5-TM-2-TA = 1,3,5-trimethyl-2-thiaadmantane; 1,3,5,7-TeM-2-TA = 1,3,5,7-tetramethyl-2-thiaadmantane; TeM-2-TA = tetramethyl-2-thiaadmantane.

In this study, 32 diamondoid compounds, including 22 adamantanes and 10 diamantanes were identified by GC–MS–MS, and their concentrations were quantified as in Table 1. Meanwhile, several homologous series of alkylated 2-thiaadamantanes were identified by GC–MS, the tentative peak assignments of alkylated 2-thiaadamantanes were given in Fig. 1.

**Table 1:** The detected diamondoid compounds in this study.

Peak number	Diamondoid compound	m/z	Abbreviation
1	Adamantane	136 → 93	A
2	1-Methyladamantane	150 → 135	1-MA
3	2-Methyladamantane	150 → 135	2-MA
4	1-Ethyladamantane	164 → 135	1-EA
5	2-Ethyladamantane	164 → 135	2-EA
6	1,3-Dimethyladamantane	164 → 149	1,3-DMA
7	1,4-Dimethyladamantane (cis)	164 → 149	1,4-DMA (cis)
8	1,4-Dimethyladamantane (trans)	164 → 149	1,4-DMA (trans)
9	1,2-Dimethyladamantane	164 → 149	1,2-DMA
10	2,6- + 2,4-Dimethyladamantane	164 → 149	2,6- + 2,4-DMA
11	1-Ethyl,3-methyladamantane	178 → 149	1-E,3-MA
12	1,3,5-Trimethyladamantane	178 → 163	1,3,5-TMA
13	1,3,6-Trimethyladamantane	178 → 163	1,3,6-TMA
14	1,3,4-Trimethyladamantane (cis)	178 → 163	1,3,4-TMA (cis)
15	1,3,4-Trimethyladamantane (trans)	178 → 163	1,3,4-TMA (trans)
16	1,2,3-Trimethyladamantane	178 → 163	1,2,3-TMA
17	1-Ethyl,3,5-dimethyladamantane	192 → 163	1-E,3,5-DMA
18	1,3,5,7-Tetramethyladamantane	192 → 177	1,3,5,7-TeMA
19	1,2,5,7-Tetramethyladamantane	192 → 177	1,2,5,7-TeMA
20	1,3,5,6-Tetramethyladamantane	192 → 177	1,3,5,6-TeMA
21	1,2,3,5-Tetramethyladamantane	192 → 177	1,2,3,5-TeMA
22	1-Ethyl-3,5,7-trimethyladamantane	192 → 177	1-E-3,5,7-TMA
I.S.-1	n-Dodecane-d26	196 → 82	n-C12-d26
23	Diamantane	188 → 131	D
24	4-Methyldiamantane	202 → 187	4-MD
25	1-Methyldiamantane	202 → 187	1-MD
26	3-Methyldiamantane	202 → 187	3-MD
27	4,9-Dimethyldiamantane	216 → 201	4,9-DMD
28	1,4-Dimethyldiamantanet 2,4-Diamethyldiamantane	216 → 201	1,4-DMD + 2,4-DMD
29	4,8-Dimethyldiamantane	216 → 201	4,8-DMD
30	3,4-Dimethyldiamantane	216 → 201	3,4-DMD
31	1,4,9-Trimethyldiamantane	230 → 215	1,4,9-TMD
32	3,4,9-Trimethyldiamantane	230 → 215	3,4,9-TMD
I.S.-2	n-Hexadecane-d34	260 → 82	n-C16-d34

### Hydrothermal experiments of HD23 oil

**Figure 2:**
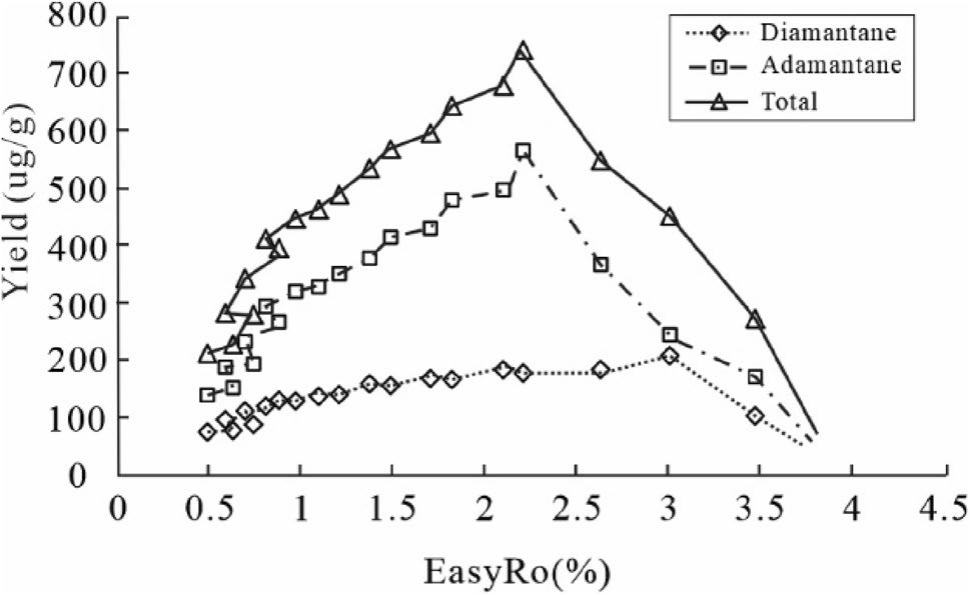
Variation in diamondoids yields (µg/g oil) with EasyRo (%) from hydrothermal pyrolysis of oil. (Total = sum of adamantanes and diamantanes).

The first sample was obtained at EasyRo = 0.48% during hydrothermal pyrolysis of an HD23 oil with yields of adamantanes and diamantanes of 137.4 µg/g and 72.4 µg/g, respectively (Supplementary Table S1 and Fig. 2). The yields of adamantanes continue to increase until EasyRo 2.1%, and at > EasyRo 2.1%, adamantanes show a decrease. The yields of diamantanes are rising from EasyRo 0.48% until EasyRo 3.0% (Fig. 2). Adamantanes dominate the generated diamondoids (Fig. 2): adamantanes have concentrations of 137.4–563.9 µg/g, which are three times more than diamantanes (from 72.4–182.1 µg/g) with the maximum value of 563.9 µg/g and 182.1 µg/g at 2.1% EasyRo and 3.0% EasyRo, respectively.

**Figure 3:**
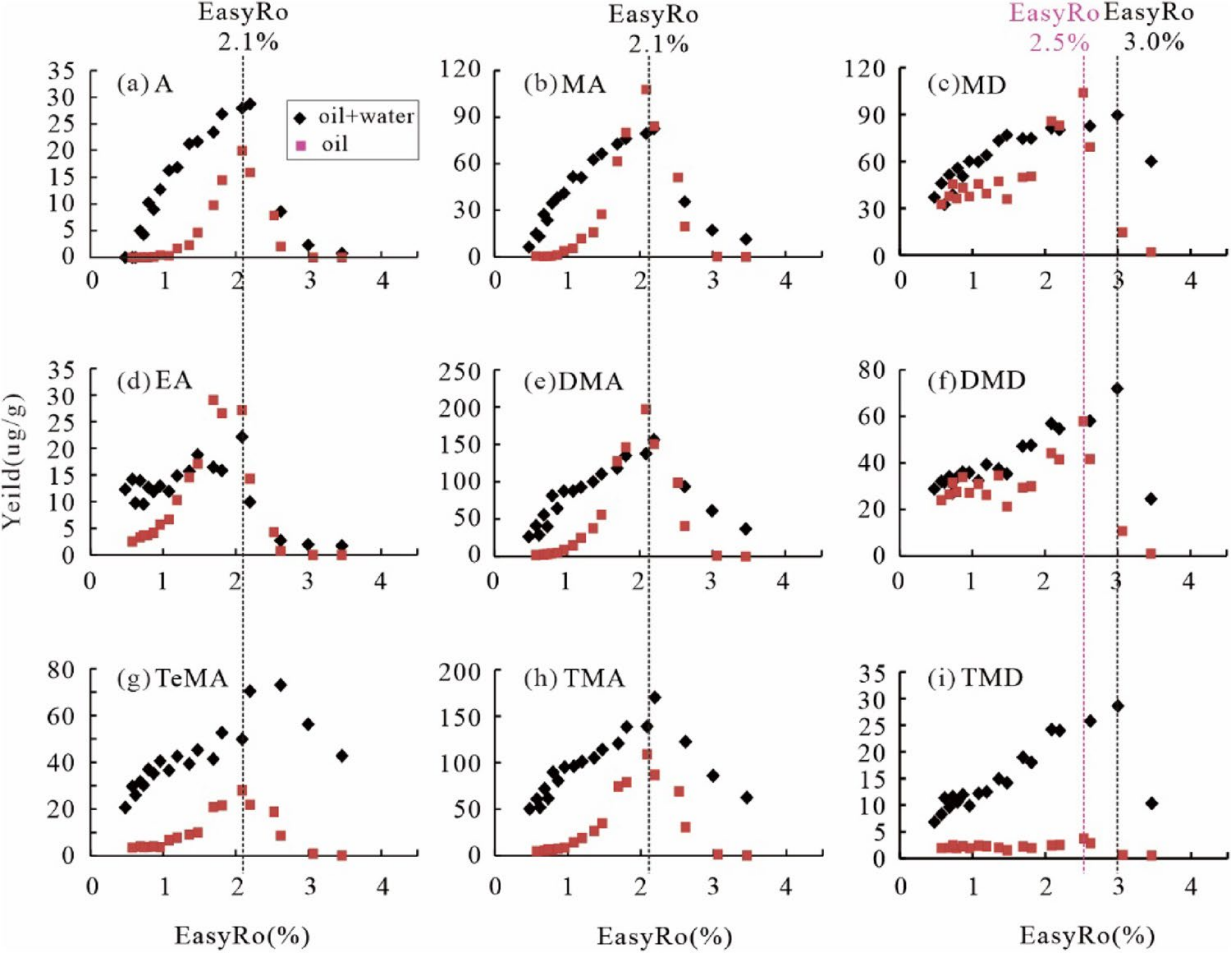
Variation in the yields (µg/g oil) of different types of diamondoids generated from hydrothermal and Fang et al.^13^ anhydrous pyrolysis of oil with EasyRo (%): (**a**) A = adamantanes, (**b**) MA = methyladamantanes, (**c**) MD = methyldiamantanes, (**d**) EA = ethyladamantanes, (**e**) DMA = dimethyladamantanes, (**f**) DMD = dimethyldiamantanes, (**g**) TeMA = Tetramethyladamantane, (**h**) TMA = trimethyladamantanes, (**i**) TMD = trimethyldiamantanes.

As for individual compounds, the amounts of generated Adamantane (A), Methyladamantane (MA), Ethyladamantane (EA), Dimethyladamantane (DMA) and Trimethyladamantane (TMA) are shown to increase with EasyRo in the range of 0.48–2.1% and rapid decrease in the EasyRo range 2.1–2.5%. The yields of Tetramethyladamantane (TeMA) increase in the EasyRo range 0.48–2.5% and a reversal occurs above the 2.5% EasyRo (Fig. 3 and Supplementary Table S1). Similarly, the yields of different types of diamantanes keep nearly constant in the oil samples from experiments at EasyRo < 1.5% (Fig. 3c,f,i). Subsequently, the yields of Methyldiamantane (MD), Dimethyldiamantane (DMD) and Trimethyldiamantane (TMD) increase in the EasyRo range 1.5–3.0% and a reversal occur above the 3.0% EasyRo (Fig. 3). In addition, adamantanes generated during oil cracking are dominated by DMA, followed by TMA, MA, TMA, EA and A, while diamantanes are dominated by MD, DMD, TMD and Diamantane (D).

### TSR experiments with ZS1-L oil

#### H_2_S and sulfur isotope data

**Figure 4:**
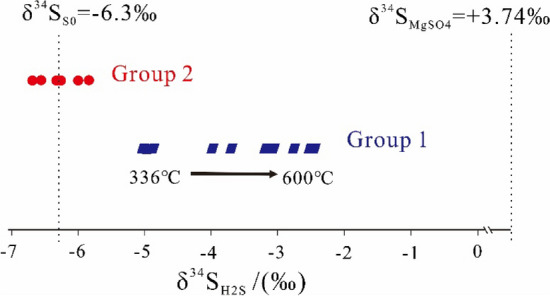
The ^34^S isotopic ratios of H_2_S in hydrothermal experiments involving S^0^, MgSO_4_ and CaSO_4_·2H_2_O.

The yields of hydrogen sulfide (H_2_S) generated in hydrothermal experiments with MgSO_4_ apparently are higher than those with CaSO_4_·2H_2_O (Table 2). For group1, the H_2_S yields increases from 9.57 mmol/g at EasyRo of 0.57 to 17.43 mmol/g at EasyRo of 2.5%, and then decreases slightly to 14.62 mmol/g at EasyRo of 3.87% (Table 2). For group 2, the H_2_S yields rise from 8.92 mmol/g to 11.47 mmol/g at EasyRo = 1.13–1.69% (Table 2). Moreover, the evolution trends for δ^34^S_H2S_ in two groups of experiments are totally different (Table 2 and Fig. 4). The δ^34^S values of group 1 H_2_S range from − 5.00‰ to − 2.45‰ with EasyRo from 0.57% to 3.87% and show a positive correlation with EasyRo (Table 2).In contrast, the δ^34^S of H_2_S generated in group 2 ranged from − 5.79‰ to − 6.79‰, within ± 1‰ of elemental S (− 6.3‰) (Table 2 and Fig. 4).

**Table 2:** Gas yields (mmol/g oil) and ^34^S isotopic ratios of H_2_S in hydrothermal experiments involving S^0^, MgSO_4_ and CaSO_4_·2H_2_O.

Temperature (℃)	Time (h)	EasyRo (%)	MgSO_4_/CaSO_4_·2H_2_O (mmol/g oil)	S^0^	H_2_S	CO_2_	H_2_S/S^0^	δ^34^S_H2S_ (‰)
**Group1#: Non-isothermal pyrolysis of ZS1-L oil involving S**^**0**^ **and MgSO**_**4**_** under constant rate of 20℃/h**								
336		0.57	13.93	13.68	9.66	0.38	0.71	− 5.00
360		0.68	13.45	12.85	9.42	0.47	0.73	− 4.87
384		0.79	13.47	12.85	9.49	0.44	0.74	− 4.93
408		0.96	13.52	12.47	10.57	0.54	0.85	− 3.96
432		1.19	13.45	12.91	11.04	0.63	0.86	− 3.68
456*		1.47	–	–	–	0.51	–	–
456		1.47	13.64	12.24	11.36	0.84	0.93	
480		1.81	13.05	12.92	12.28	1.07	0.95	− 3.05
504		2.19	11.41	11.59	15.12	1.15	1.31	− 2.74
528		2.62	12.86	12.79	17.43	1.70	1.36	–
552		3.06	13.84	13.00	16.96	1.93	1.30	− 2.97
576		3.5	13.35	12.18	15.58	1.99	1.28	− 2.49
600		3.87	13.41	13.11	14.62	2.14	1.12	− 2.45
**Group2#: Isothermal pyrolysis of ZS1-L oil involving S**^**0**^** and CaSO**_**4**_·**2HO at 360℃ for 48–840 h2**								
360*	432	1.54	–	–	–	1.23	–	–
360	48	1.13	13.55	13.09	11.46	1.71	0.88	–
360	96	1.25	13.79	12.38	10.53	1.75	0.93	–
360	144	1.32	13.70	13.45	11.47	1.75	0.85	− 5.97
360	192	1.38	13.33	12.39	8.92	1.74	0.78	− 6.20
360	288	1.46	13.75	13.74	9.48	1.99	0.69	− 5.78
360	432	1.54	13.79	13.36	10.26	2.21	0.71	− 6.28
360	648	1.63	13.70	13.25	9.25	2.21	0.65	− 6.79
360	840	1.69	13.63	14.18	11.14	2.49	0.73	− 6.79

–Indicates not detected; *The blank experiments.

#### Diamondoids and thiadiamondoids data

**Figure 5:**
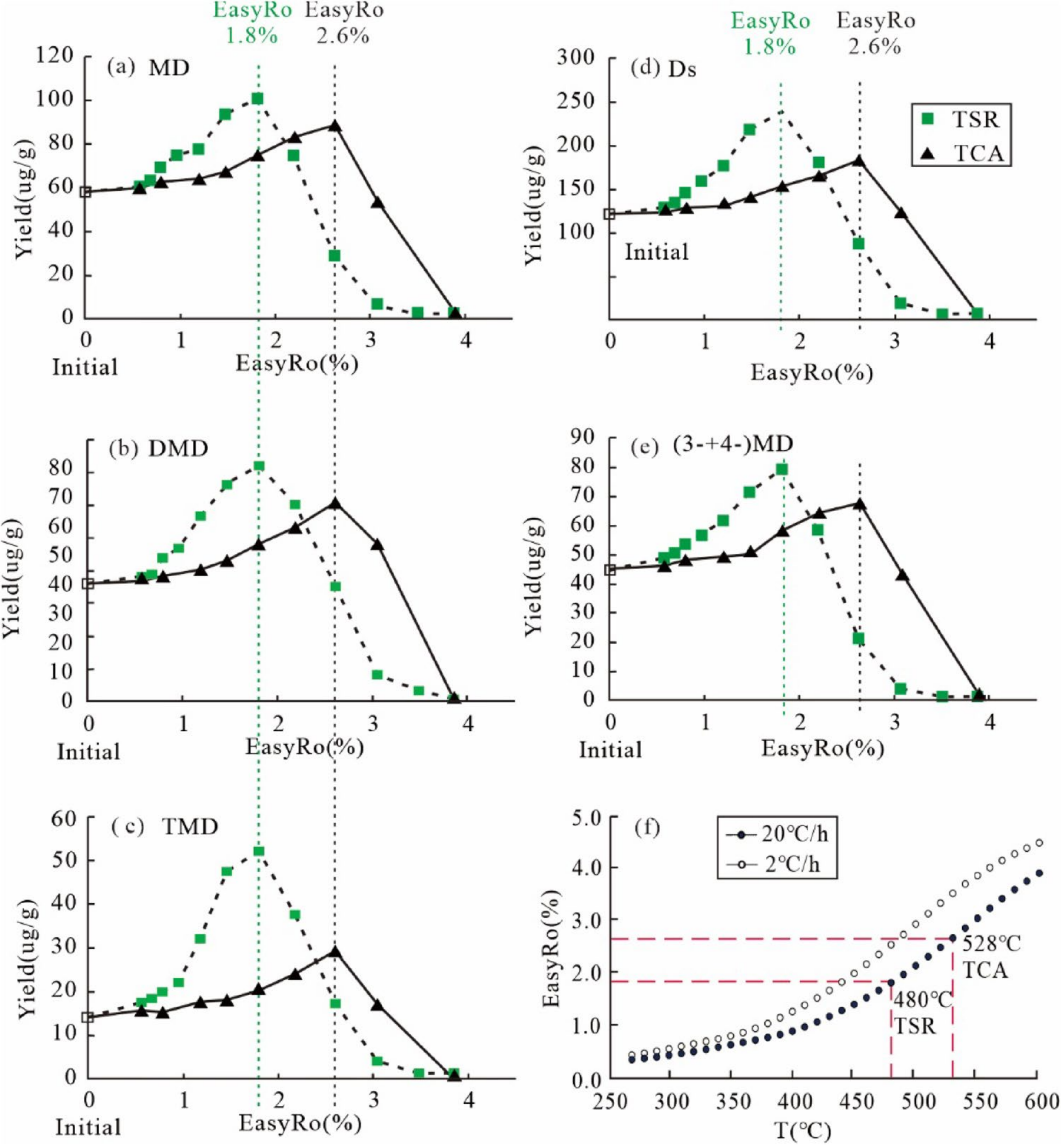
Plots showing the correlation of EasyRo (%) or heating temperature (°C) with the yields of different types of diamantanes (µg/g oil) in the TSR experiments (Group1) and TCA experiments: (**a**) MD versus EasyRo, (**b**) DMD versus EasyRo, (**c**) TMD versus EasyRo; (**d**) Ds versus EasyRo; (**e**) (3- + 4-)MD versus EasyRo (**f**) heating temperature versus EasyRo. MD = methyldiamantanes; DMD = dimethyldiamantanes; TMD = trimethyldiamantanes; Ds = total diamantanes.

For the yields of diamondoids, only diamantanes from the TSR experiments in group 1 are discussed in this study. See “Hydrothermal experiments of HD23 oil” section for details. Adamantanes were evaporatively lost during sample working up, the collected samples show elevated diamantanes yields, and thus only results of diamantanes are listed (Table 3). The yields of total diamantanes and (3- + 4-) MD progressively rises from 129.58 µg/g and 49.11 µg/g before the heating to a maximum of 249.15 µg/g and 79.45 µg/g at EasyRo 1.81%, respectively (Fig. 5d,e). At EasyRo > 1.81%, both (3- + 4-) MD and diamantanes show a decrease. Diamantanes generated during TSR are dominated by MD, followed by DMD, TMD and D (Fig. 5a–c). Interestingly, thiadiamondoids including thiaadmantane and methyl thiaadmantanes isomers were detected from the oil after TSR pyrolysis in the 480 °C experiments (1.81% EasyRo) with the maximum yield of diamantanes (Fig. 1).

**Table 3:** The yields (µg/g oil) of individual diamantane compounds identified in Table 1 at each heating temperature of TSR experiments (Group1) and TCA experiments.

EasyRo	T (20 °C /h)	D	4-MD	1-MD	3-MD	4,9-DMD	1,4- + 2,4-DMD	4,8- DMD	3,4- DMD	1,4,9-TMD	3,4,9-TMD	MD	DMD	TMD	3- + 4-MD	Ds	MDI	DMDI-1	DMDI-2
0	initial	14.08	20.92	13.28	23.89	5.98	6.46	8.00	14.90	3.22	10.98	58.09	35.34	14.20	44.81	121.72	0.36	0.29	0.43
**TSR experiments: ZS1-L oil** + **S**^**0**^ + **MgSO**_**4**_ +** Water solution**^**a**^																			
0.57	336	13.86	24.30	11.42	24.81	7.09	6.36	6.66	17.43	4.35	13.28	60.53	37.55	17.63	49.11	129.58	0.40	0.29	0.52
0.68	360	15.16	24.46	13.08	25.79	7.60	6.70	7.09	16.83	4.69	13.82	63.32	38.22	18.51	50.24	135.22	0.39	0.31	0.52
0.79	384	13.94	25.79	15.54	27.96	7.48	6.91	8.15	20.57	4.79	15.24	69.29	43.11	20.03	53.75	146.37	0.37	0.27	0.48
0.96	408	15.66	26.31	18.26	30.31	8.97	7.68	7.66	21.82	5.34	16.87	74.87	46.13	22.21	56.61	158.87	0.35	0.29	0.54
1.19	432	11.26	17.46	15.82	44.35	8.95	10.26	10.17	26.52	7.73	24.47	77.63	55.90	32.19	61.81	176.98	0.22	0.25	0.47
1.47	456	12.27	18.57	22.03	53.08	9.13	9.50	11.49	35.28	7.77	39.84	93.67	65.40	47.61	71.64	218.95	0.20	0.21	0.44
1.81	480	14.84	23.24	21.45	56.21	10.98	11.99	13.40	34.77	9.10	43.18	100.89	71.14	52.28	79.45	239.15	0.23	0.24	0.45
2.19	504	9.96	18.21	16.10	40.42	8.55	8.47	9.78	32.65	6.65	31.13	74.74	59.45	37.78	58.63	181.92	0.24	0.21	0.47
2.62	528	7.49	10.32	7.46	11.05	8.27	6.62	7.27	12.28	7.20	10.13	28.83	34.45	17.33	21.37	88.10	0.36	0.40	0.53
3.06	552	2.33	2.31	1.73	1.87	1.75	1.73	1.73	2.37	1.76	2.37	5.92	7.59	4.13	4.18	19.97	0.39	0.43	–
3.5	576	0.75	0.70	0.70	0.70	0.70	0.70	0.70	0.70	0.70	0.70	2.10	2.80	1.40	1.40	7.05	–	–	–
3.87	600	1.00	0.75	0.75	0.75	0.75	0.75	0.75	0.75	0.75	0.75	2.25	3.00	1.50	1.50	7.76	–	–	–
**TCA experiments: ZS1-L oil** + **Water solution**^**b**^																			
0.57	336	13.40	18.23	13.30	27.27	5.96	7.49	7.89	15.78	4.05	11.74	58.80	37.12	15.79	45.49	125.11	0.31	0.27	0.43
0.79	384	12.80	18.96	13.98	28.67	6.00	7.52	8.03	16.07	4.09	11.31	61.61	37.62	15.40	47.62	127.43	0.31	0.27	0.43
1.19	432	12.67	19.33	14.35	29.44	6.04	8.07	8.04	17.48	4.15	13.57	63.12	39.63	17.72	48.78	133.15	0.31	0.26	0.43
1.47	456	13.37	18.89	15.63	31.90	6.33	8.93	8.00	19.11	4.07	14.15	66.42	42.38	18.21	50.79	140.38	0.28	0.25	0.44
1.81	480	11.01	21.02	16.13	37.08	7.63	9.34	8.08	22.36	4.48	16.35	74.22	47.41	20.83	58.10	153.46	0.28	0.25	0.49
2.19	504	6.83	21.19	18.36	43.22	8.09	9.72	9.73	24.90	5.15	19.05	82.77	52.44	24.20	64.42	166.25	0.26	0.25	0.45
2.62	528	7.09	23.25	20.56	44.63	8.84	10.78	11.48	28.91	6.17	23.22	88.44	60.02	29.40	67.88	184.95	0.26	0.23	0.44
3.06	552	4.97	18.90	9.82	23.54	8.69	9.42	9.04	20.32	6.04	11.12	52.26	47.46	17.16	42.44	121.85	0.36	0.30	0.49
3.87	600	–	–	–	–	–	–	–	–	–	–	–	–	–	–	–	–	–	–

–indicates not detected.

^a^Water solution containing: 5.6 wt.% MgCl_2_, 10 wt.% NaCl and 0.56 wt.% MgSO_4_.

^b^Water solution containing: 5.6 wt.% MgCl_2_, 10 wt.% NaCl.

In the non-TSR or TCA experiments (blank experiments), the maximum yields of total diamantanes and (3- + 4-) MD were 184.95 µg/g and 67.88 µg/g, which are significantly lower than those from TSR experiments, respectively (Fig. 5d,e). In addition, an obvious lag in reversals (2.62% EasyRo) occurred for non-TSR experiments compared to TSR (1.81% EasyRo). Similar to the TSR experiments, diamantanes generated during TCA are dominated by MD, followed by DMD, TMD and D (Fig. 5a–c).

## Discussion

### Formation and decomposition of diamondoids during hydrothermal pyrolysis of an HD23 oil

**Figure 6:**
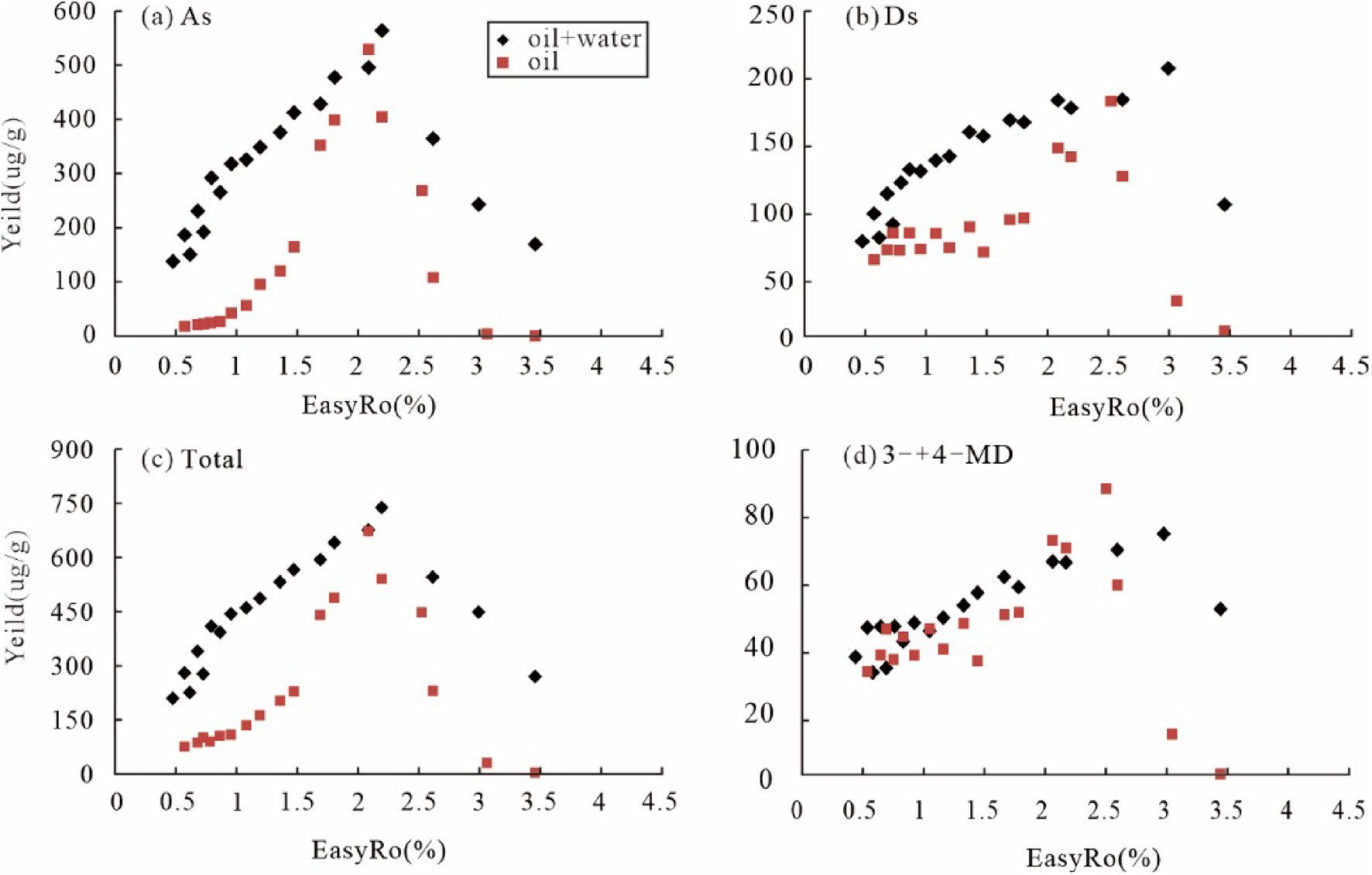
Variation in the yields (µg/g oil) of diamondoids generated from hydrothermal and Fang et al.^13^ anhydrous pyrolysis of the same oil with EasyRo (%): (**a**) As, (**b**) Ds, (**c**) Total, (**d**) (3- + 4-) MD. As = total adamantanes; Ds = total diamantanes.

Hydrothermal pyrolysis of the HD23 oil shows that both adamantanes and diamantanes were newly generated and decomposed. Still, their yield curves are partially different from the anhydrous^13^: First, diamondoids were generated in a broader range of EasyRo with higher yields at < 1.7% EasyRo during the hydrothermal experiments than the anhydrous (Fig. 6), indicating that water promoted the yields of diamondoids at low EasyRo (< ~ 2.0%). With increasing EasyRo, the differences in the yields of diamondoids between the two became smaller. Among diamondoids, adamantanes show an increase in their yields from 0.48% to 2.1% EasyRo (Fig. 6a), and the range is wider than the range of 1.0–2.1% for the anhydrous experiments. Similarly, diamantanes began to be generated at 0.79% EasyRo from hydrothermal pyrolysis experiments, much lower than 1.7% EasyRo for the anhydrous pyrolysis experiments (Fig. 6b). Second, the decomposition of diamantanes and TeMA from the hydrothermal experiments occurred at EasyRo > 3.0% and EasyRo > 2.5%, obviously lagging behind that from the corresponding anhydrous experiments at EasyRo > 2.5% and 2.1%, respectively (Figs. 3 and 6b). This may indicate that water can delay the decomposition of high molecular weight diamondoids during oil thermal cracking.

Larger yields of diamondoids from hydrothermal pyrolysis than the anhydrous (Fig. 6) can be explained as follow. As the result of ionic reactions, hydrothermal pyrolysis of organic matter generates more considerable amounts of intermediate olefinic and isomeric hydrocarbons than the anhydrous pyrolysis^53^. In turn, the olefins and isomeric hydrocarbons will be hydrogenated by rapid free radical reactions, raising the yields of diamondoids during hydrothermal pyrolysis. That is, combining ionic and free radical reactions can accelerate isomerization and cyclization of these olefinic hydrocarbons to generate the relatively high yields of diamondoids under hydrothermal pyrolysis.

It is necessary to discuss which one, hydrothermal or anhydrous pyrolysis, has the products representing maturation of natural samples, considering the more significant differences in EasyRo for decomposition of diamantanes and yields of diamondoids between the two. The EasyRo for the generation and decomposition of the (3- + 4-) MD in this study are close to that of natural samples from both coals and rocks, that is, ca. 1.2% EasyRo vs 1.1% Ro for the generation and 3.0% EasyRo vs ca. 4.0% Ro for the decomposition^7^. In contrast, EasyRo obtained from anhydrous pyrolysis are deviated more from the natural samples, 1.5% for the generation and 2.5% for the decomposition^13,54,55^. Ro values are approximately equal to the calculated EasyRo values at EasyRo < 1.5 ~ 2.0%. The differences between Ro and calculated EasyRo are slightly more significant at EasyRo > 1.5 ~ 2.0%, likely due to the change in the chemical composition of solid kerogen with higher maturity levels^56^. This result suggests that hydrothermal pyrolysis has the products closer to the cracking of natural samples, which is supported by the gas produced from the hydrothermal pyrolysis more similar to the natural gas than anhydrous pyrolysis^23^. Moreover, water is ubiquitous in petroleum reservoirs and may provide H and O involved in petroleum generation and evolution^42,57^, suggesting hydrothermal pyrolysis may represent the maturation of natural samples better than the anhydrous.

### Diamondoids as proxies for thermal maturity

**Figure 7:**
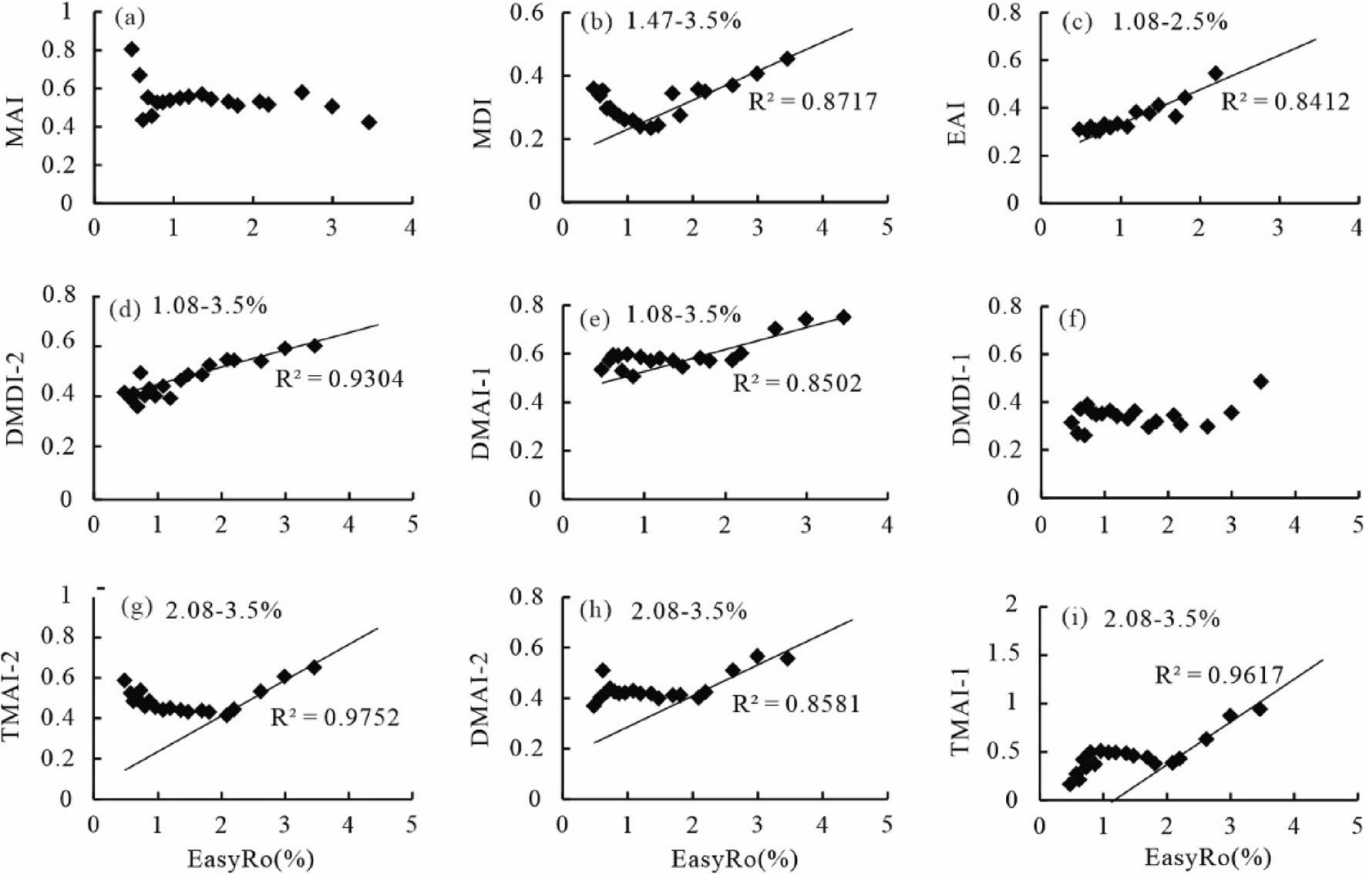
Plots showing the variation of diamondoid indices (MAI, MDI, DMAI-1, DMAI-2, DMDI-1, EAI, TMAI-1 and TMAI-2) with EasyRo (%) from anhydrous and hydrothermal pyrolysis of oil. MAI = 1-MA/(1-MA + 2-MA), MDI = 4-MD/(4-MD + 1-MD + 3-MD), DMAI-1 = 1,3-DMA/(1,2-DMA + 1,3-DMA), DMAI-2 = 1,3-DMA/(1,2-DMA + 1,4-DMA), DMDI-1 = 3,4-DMD/(4,9-DMD + 3,4-DMD), DMDI-2 = 4,8-DMD/(4,9-DMD + 4,8-DMD), EAI = 2-EA/(1-EA + 2-EA), TMAI-1 = 1,3,5-TMA/(1,3,5-TMA + 1,3,4-TMA), TMAI-2 = 1,3,5-TMA/(1,3,5-TMA + 1,3,6-TMA).7.

It is widely accepted those isomerization ratios such as MAI. MDI, EAI, DMAI-1, TMAI-1, TMAI-2, DMDI-1 and DMDI-2 can be used to determine the thermal maturity of highly mature crude oils (Ro > 1.1%)^9,10,30,32,34^, and they can be applied for different maturity ranges^16^. Isomerization-related diamondoid ratios are unaffected by thermal maturity levels with EasyRo < 2.0% in anhydrous pyrolysates and used as proxies of thermal maturity at > 2.0% EasyRo^16^. In this study, MDI, EAI, DMAI-1 and DMDI-2 can be applied to reflect maturity at much lower EasyRo from hydrothermal pyrolysis: 1.47–3.5% EasyRo for MDI with R^2^ of 0.8717 (Fig. 7b), 0.86–2.5% EasyRo for EAI with R^2^ of 0.8412 (Fig. 7c), 1.08–3.5% EasyRo for DMAI-1 with R^2^ of 0.8502 (Fig. 7e) and 1.08–3.5% EasyRo for DMDI-2 with R^2^ of 0.9304 (Fig. 7d). This supports that MDI is an effective proxy of maturity at > 1.3% Ro for either source rock extracts^9^ or hydrothermal pyrolysates^10^. However, consistent with Fang et al.^13^, MAI in this study seems not related to EasyRo (Fig. 7a), and thus cannot be used as a proxy to assess the thermal maturity of oils. MDI, EAI, DMAI-1, DMDI-2 can serve as reliable maturity indicators with broad EasyRo ranges mainly > 1.0%. In contrast, at EasyRo < 1.0%, diamondoid-related proxies including MDI, EAI, DMAI-1, DMDI-2 show no correlations with EasyRo, suggesting that they cannot be used to determine the maturity of oils and thus source rocks. The previous observation supports this proposal that diamondoid concentrations and distributions are dependent on the source rocks instead of maturity within the oil window^58^.

Other isomerization ratios (e.g., DMAI-2, TMAI-2 and TMAI-1) show good correlations with thermal maturity in the higher EasyRo ranges of 2.08–3.5% with R^2^ of 0.9617, 0.9752 and 0.8581 (Fig. 7g–i). These ratios seem controlled by the parent organic matter during the generation stage of diamondoids (EasyRo < 2.0%), and thus may reflect the source feature rather than maturity^16^. They can be used to reflect maturity only at higher maturity levels (> 2.0% EasyRo) as found in Fang et al.^16^ and this study. However, unlike other studies, DMDI-1 does not correlate well with EasyRo values in this study (Fig. 7f), probably due to the relatively sizeable analytical error associated with low concentrations of dimethyldiamantanes in the pyrolysates.

### Diamondoids as proxies for the extent of oil cracking

**Figure 8:**
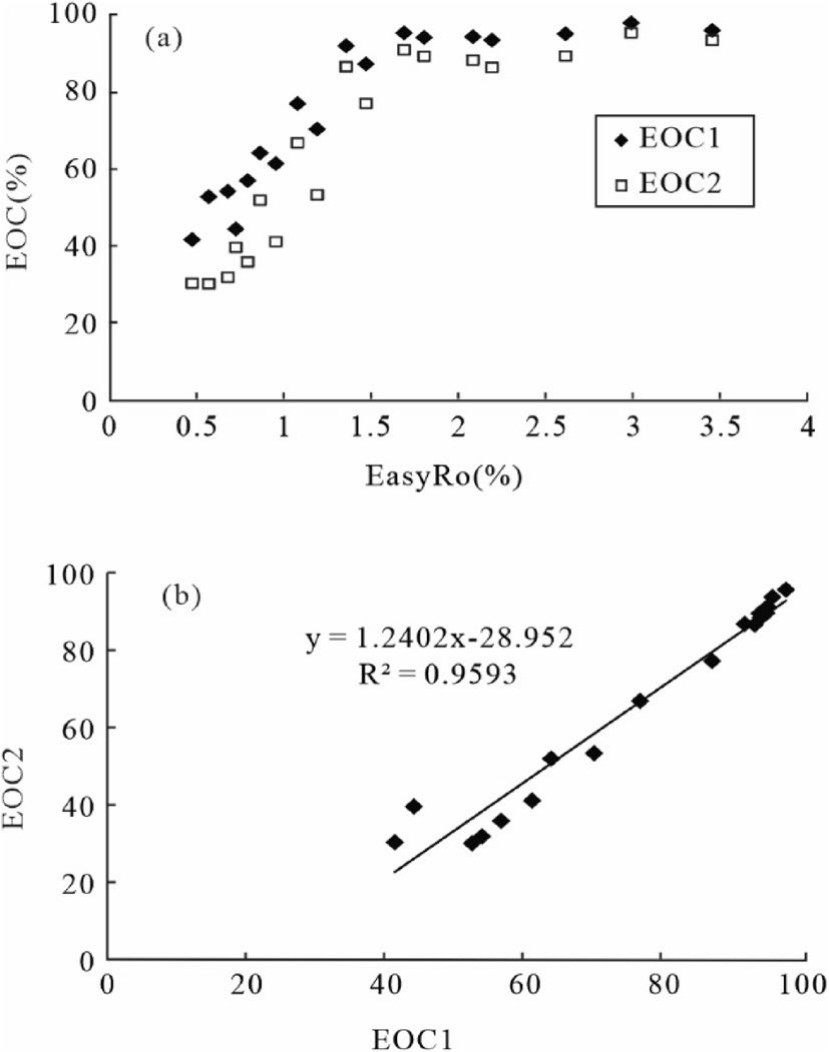
Relationships between: (**a**) EOC (%) and EasyRo (%), (**b**) the actual EOC2 (%) and the calculated EOC1 (%). EOC1: the calculated EOC (%) from (1 − C_0_/C_c_) × 100; EOC2: the actual EOC (%).

Oil cracking involves the thermal breakdown of heavy hydrocarbons to smaller ones, or the process of ultimately converting oil to hydrogen-rich gas and carbon-rich pyrobitumen^59^. In our hydrothermal pyrolysis, we found that the extent of oil cracking (EOC; i.e., the percentage of liquid hydrocarbon converted to gas and pyrobitumen, or *EOC* = *(1-M*_*c*_*/M*_0_*)* × *100*, M_c_ and M_o_ are residual and initial liquid hydrocarbons, respectively) can rapidly increase to 90% with the rise in EasyRo from 0.48% to 1.81% (Fig. 8a). Oil cracking occurs at slower rates with further increasing maturation as reflected in the increase in EasyRo from 1.81% (480 °C) to 3.5% (600 °C) and relatively stable EOC around 90% to 95%. However, at the high maturity (above 500 °C) almost all of the liquid hydrocarbons have been consumed, so the error is around ± 5% from 2.19% (504 °C) to 3.5% (600 °C) in the oil pyrolysis experiments.

**Figure 9:**
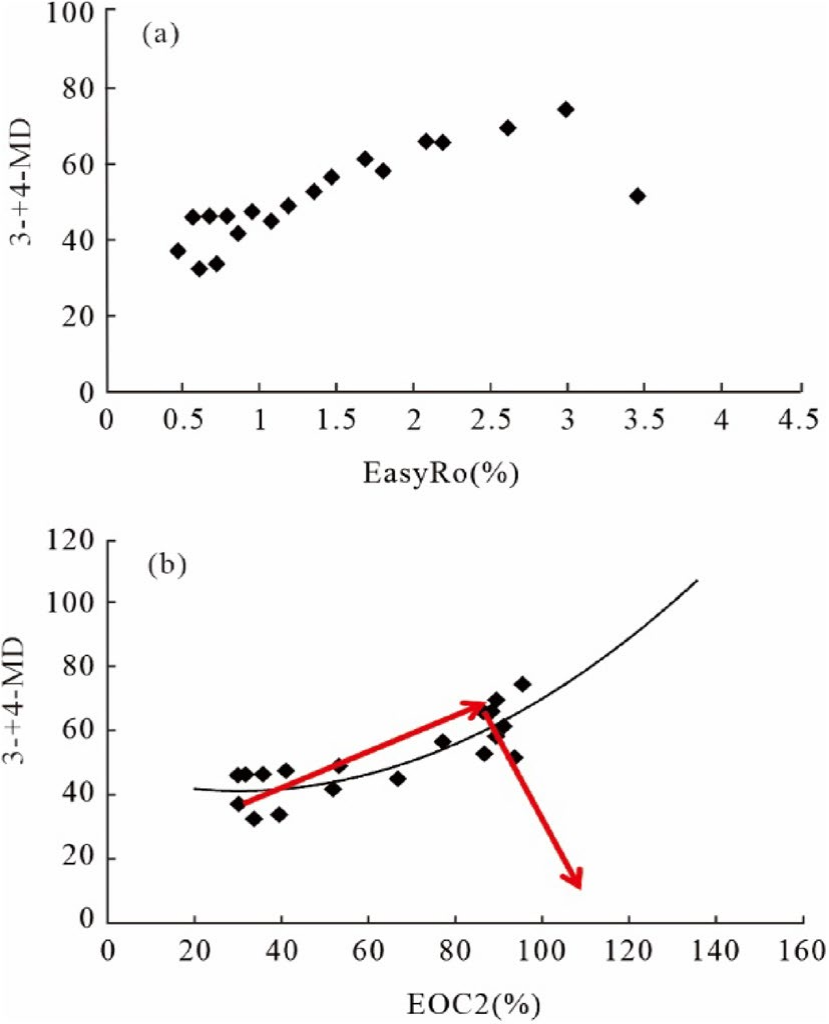
Relationships between: (**a**) the yield of (3- + 4-) MD and EasyRo (%), (**b**) the yield of (3- + 4-) MD and EOC (%).

EOC can also be calculated as (1 − C_0_/C_c_) × 100^5^, in which (3- + 4-) MD is assumed not to have newly been generated or decomposed during oil cracking (C_0_ and C_c_ are concentrations of (3- + 4-) MD before and after oil cracking). However, an increase in the (3- + 4-) MD occurs at ca. 1.2% EasyRo. The decrease in the (3- + 4-) MD yield is observed at 3.0% EasyRo during oil thermal cracking experiments (Fig. 9a), suggesting the assumption does not apply (Fig. 9b). This finding is supported by other pyrolysis experiments^8,13,16^, lending usage of *(1-C*_*0*_*/C*_*c*_*)* × *100%* is suspect. Based on our results, Dahl’s formula for EOC is only applicable to a very narrow range of maturity (EasyRo < 1.2%), and gives higher values than those obtained from our hydrothermal experiments (Fig. 8a). The differences between the two results become progressively smaller with the increasing extent of oil cracking with the values from 6 to 21% at EasyRo from 0.48% to 1.81% and from 2.5% to 6% at EasyRo from 1.81 to 3.0%. Obviously, the *(1-C*_*0*_*/C*_*c*_*)* × *100%* should be changed to *[1-C*_*0*_*/(C*_*c*_*-C*_*new gener*_*)]* × *100%* at 1.2% < EasyRo < 3.0%, but the C_new gener_ is difficult to obtain. Fortunately, we find that the calculative EOC (*EOC1* = *[1 − C*_*0*_*/C*_*c*_*]* × *100*) shows a good positive linear correlation with the actual EOC (*EOC2* = *[1-M*_*c*_*/M*_*0*_*]* × *100*) with equation of EOC2 = 1.2402 × EOC1 − 28.952 and R^2^ value of 0.9593 at EasyRo < 3.0% (Fig. 8b). This reveals that although Dahl’s method may overestimate the extent of oil cracking, especially in highly cracked samples due to the new generation of 3- + 4-MD, the method can be corrected and new calculation formula can be used to reflect actual EOC.

**Figure 10:**
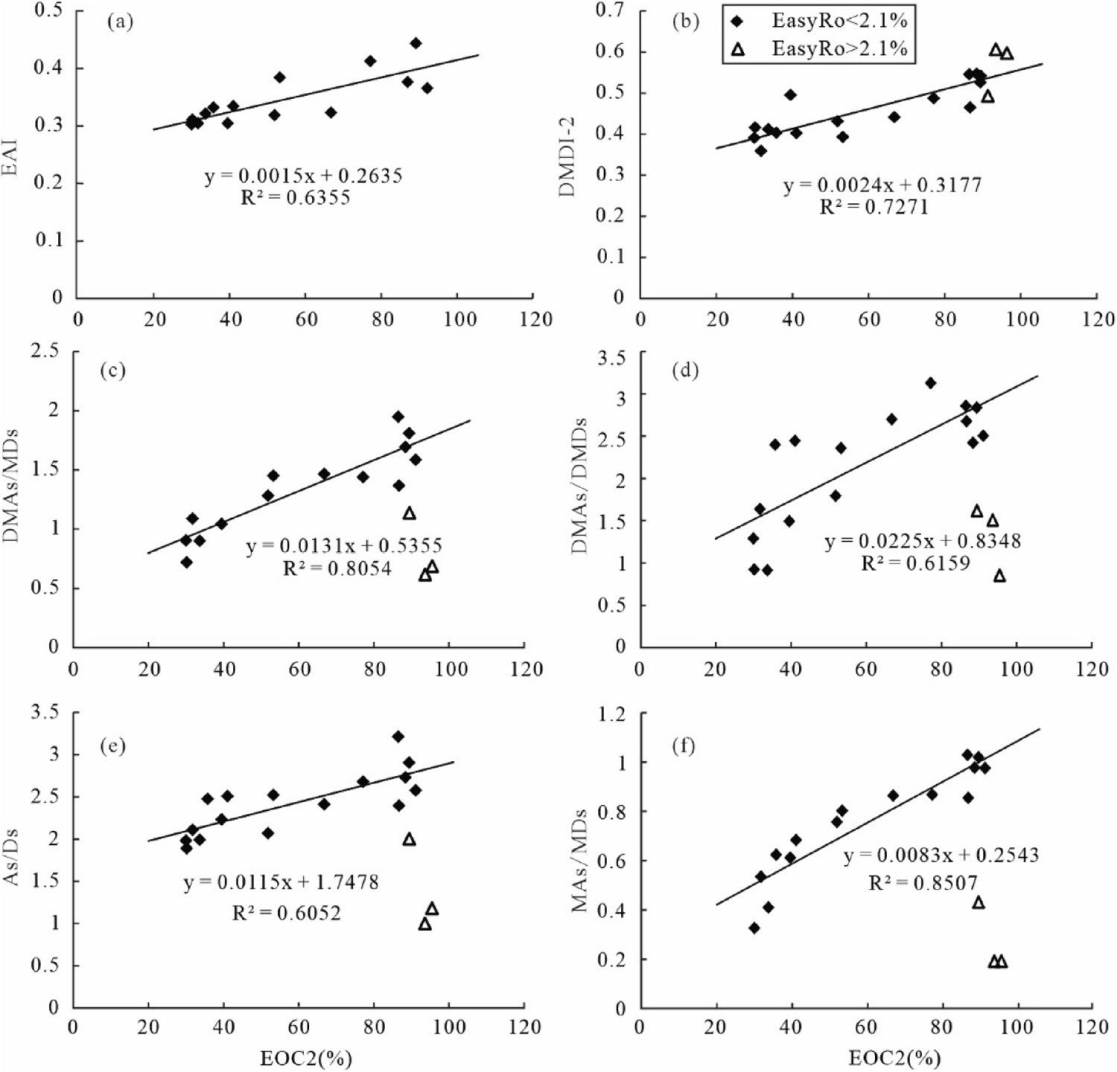
Relationships between diamondoid-related proxies and EOC2 (%): (**a**) EAI; (**b**) DMDI-2; (**c**) DMAs/MDs, (**d**) DMAs/DMDs, (**e**) As/Ds, (**f**) MAs/MDs. Triangles indicate data from EasyRo > 3.0%.

The bridgehead-methylated diamondoids are thermodynamically more stable than other methylated diamondoid species^33^. On this basis, some diamondoid isomerization ratios (MAI, MDI, DMAI-1, DMAI-2, TMAI-1, TMAI-2, EAI, DMDI-1, DMDI-2) are used as maturity indicators. Figure 10a,b shows that there is a good positive correlation between diamondoid isomerization ratios (EAI and DMDI-2) and EOC2 with regressive equations as follow, where EAI is applicable in the range of EasyRo < 1.81% (Fig. 10a).

2 $${\text{EAI}} = 0.0015\;{\text{EOC}}2 + 0.2635 \quad \quad {\text{r}}^{2} = 0.6355\;\left( {{\text{EasyRo}} < 1.81\% } \right)$$

3 $${\text{DMDI-}}2 = 0.0024\;{\text{EOC}}2 + 0.3177\quad \quad {\text{r}}^{2} = 0.7271$$

This implies that these parameters might help assess the extent of oil cracking (EOC2). Note that the isomerization ratio of DMDI-2 has a good correlation with EOC2 throughout the EasyRo range examined, indicating that it may be a reliable proxy for a wide range of maturity.

On the other hand, the concentration ratios of diamondoid pairs are expected to eliminate the effect of matrix changes during the thermal cracking of oil. Some diamondoid concentration ratios (As/Ds, MAs/MDs, DMAs/DMDs, and DMAs/MDs) appear positively correlated with EOC2 at EasyRo from 0.48% to 2.1% (Fig. 10c-f) with regressive equations as follow.

4 $${\text{As/Ds}} = 0.0115\;{\text{EOC}}2 + 1.7478 \quad \quad {\text{r}}^{2} = 0.6052\;\left( {{\text{EasyRo}} < 2.1\% } \right)$$

5 $${\text{MAs/MDs}} = 0.0083\;{\text{EOC}}2 + 0.2543 \quad \quad {\text{r}}^{2} = 0.8507\;\left( {{\text{EasyRo}} < 2.1\% } \right)$$

6 $${\text{DMAs/MDs}} = 0.0131\;{\text{EOC}}2 + 0.5355 \quad \quad {\text{r}}^{2} = 0.8054\;\left( {{\text{EasyRo}} < 2.1\% } \right)$$

7 $${\text{DMAs/DMDs}} = 0.0225\;{\text{EOC}}2 + 0.8348 \quad \quad {\text{r}}^{2} = 0.6159\;\left( {{\text{EasyRo}} < 2.1\% } \right)$$

However, the above diamondoid isomerization ratios negatively correlate with EasyRo values of > 2.1% when admantanesadamantanes enter the decomposition stage. The above equations established from hydrothermal pyrolysis are proposed to be used as proxies of the extent of oil cracking during 0.48–2.1% EasyRo for natural petroleum reservoirs.

### New generation of diamondoids and thiadiamondoids during TSR?

#### Occurrence of TSR in the experiments

In group 1 and group 2 experiments, H_2_S may have been derived from: 1) cracking of ZS1-L oil, 2) elemental sulfur hydrolysis, 3) thermochemical reduction of MgSO_4_ or CaSO_4_·2H_2_O. The H_2_S is not mainly from cracking of ZS1-L oil because thermal decomposition of ZS1-L oil with a sulfur content of 0.18% can only generate 0.056 mmol/g H_2_S. Thus, H_2_S must have mainly derived from the reduction of elemental S, MgSO_4_ or CaSO_4_·2H_2_O. Elemental S may react with water at temperatures as low as 200 °C in the following disproportionation reaction^51,60,61^:

8 $$4{\text{S}}^{0} + 4{\text{H}}_{2} {\text{O}} \to {\text{SO}}_{4}^{2 - } + 3{\text{H}}_{2} {\text{S}} + 2{\text{H}}^{ + }$$

An alternative production pathway for the exceptionally high yields of H_2_S was via the classical aqueous reaction of elemental S and hydrocarbon shown in Eq. (9)^17,26^:

9 $$4{\text{S}}^{0} + 1.33({-\!\!-}{\text{CH}}_{2} {-\!\!-}) + 2.66{\text{H}}_{2} {\text{O}} \to 4{\text{H}}_{2} {\text{S}} + 1.33{\text{CO}}_{2}$$

The H_2_S/S^0^ molar ratio can be used to determine the amount of H_2_S from the conversion of elemental S (Table 2), and thus will approach 0.75 and 1 for Eqs. (8) and (9), respectively.

The results from group 1 experiment at the lowest temperature of 336 °C (0.57% EasyRo) has H_2_S/S^0^ molar ratio of around 0.71 and δ^34^S_H2S_ value of − 5‰ (Table 2), being close to that of elemental S (-6.3‰),indicating that nearly all H_2_S was derived from elemental S. Furthermore, the increasing production of CO_2_ did not start until above 408 °C, when the H_2_S/S^0^ molar ratio began to be greater than 0.75. This inconsistency in the product implies H_2_S was not produced by elemental sulfur via Eq. (8) from 336–384 °C (0.57–0.79% EasyRo) in group 1. Thus, MgSO_4_ source has to be considered with an Eq. (10).

10 $${\text{MgSO}}_{4} + 2{\text{H}}^{ + } + {\text{CH}}_{4} \to {\text{CO}}_{2} + {\text{H}}_{2} {\text{S}} + 2{\text{H}}_{2} {\text{O}} + {\text{Mg}}^{2 + }$$

It can be expected that with an increase in temperature, more MgSO_4_ is involved in TSR reaction, or TSR proceeds to higher degrees. Suppose all H_2_S was generated from reactions of S^0^ with hydrocarbons without generation of SO_4_^2−^ (Eq. 9). In that case, the H_2_S is expected to have the molarity of elemental S of about 12.79 mmol (26.8 mg), which is lower than H_2_S from experiments at 504 °C to 600 °C. Hence, the conversion of elemental S is insufficient for the generation of H_2_S from 504 °C to 600 °C, suggesting that MgSO_4_ in group 1 experiments must have beeninvolved in the reaction. On the other hand,the H_2_S/S^0^ molar ratio gradually increases until it reaches a maximum of 1.36 at 528 °C (2.62% EasyRo) with the temperature increasing, then gradually decreases to 1.12 (Table 2). Meanwhile, the δ^34^S value of H_2_S show rise from − 5‰ to − 2.45‰, getting closer to the δ^34^S value of MgSO_4_ (δ^34^S of + 3.75‰), suggesting that the H_2_S may have significantly derived from the reduction of MgSO_4_ in the aqueous experiments following Eq. (10).

Note that some of the H_2_S is expected to react with hydrocarbons to form OSCs such as thiols, (poly)sulfides, thiophenes, and benzothiophenes^62,63^, thus free H_2_S amount is expected to be lower than that of decrease in reactants MgSO_4_ and elemental sulfur and more incorporation may have occurred and thus shows a decreasing trend at EasyRo = 2.5–3.87% (Table 2).

In contrast, no TSR may have occurred in group 2 experiments but reactions between elemental S and hydrocarbons with no CaSO_4_·2H_2_O involved. Firstly, the maximum value of the H_2_S/S^0^ molar ratio in Group 2 is around 0.9 at the first EasyRo = 1.13 and then slightly decreases from 0.93 to 0.65 with EasyRo from 1.13% to 1.69%. Secondly, the δ^34^S of H_2_S generated in Group 2 ranged from − 5.79‰ to − 6.79‰, within ± 1‰ of elemental S (-6.3‰). Finally, Group 2 produced a very high amount of CO_2_ (1.71 mmol/g at 1.13% EasyRo) at the first desired time compared to the meager yields produced by Group 1 above 360 °C (Table 2). This indicates H_2_S was generated via the classical aqueous reaction of elemental S and hydrocarbon shown in Eq. (9).

Therefore, it can be concluded that TSR has occurred in the group 1 experiments as reflected by the positive shift in δ^34^S value of H_2_S due to reactant MgSO_4_ as the most ^34^S-enriched sulfur species in this study,the increase of CO_2_ and H_2_S/S^0^ molar ratio, and shows higher degrees with increasing temperatures. In contrast, the non-TSR reactions between hydrocarbons and H_2_S or elemental sulfur as shown by group 2 experiments have produced H_2_S/S^0^ molar ratio of 0.75 and δ^34^S value of H_2_S close to the elemental sulfur.

#### Generation of diamondoids during TSR

Group 1 experiments show the presence of TSR reaction significantly accelerates the generation and increases the yield of diamantanes relative to the blank non-TSR experiments (TCA; Fig. 5). Here, diamantanes are shown to be predominantly generated during TSR in the EasyRo range of 0.57–1.81% with maximum yields of 240 μg/g at 1.81% EasyRo. However, peak generation of diamantanes of 184.9 μg/g from TCA on ZS1-L oil occurs at 2.62% EasyRo (Fig. 5d), which is significantly lower than TSR (Fig. 5d). In addition, diamantanes remain stable at up to 528 °C during TCA while the temperature is 480 °C during TSR at the same heating rates of 20 °C /h (Fig. 5f). This result may be due to the catalysis of S radical (i.e., from H_2_S), which can accelerate the decomposition of HC or OM.

Moreover, TSR significantly increases the yield of diamantanes compared with the thermal chemical alteration (TCA; Fig. 5a–e). From 0.57% EasyRo to 1.81% EasyRo, the yield of diamantanes detected in the TSR was higher than that of TCA, indicating that diamantanes must have been newly generated during TSR (Fig. 5d). Elemental S can substantially lower the onset temperature of thermal chemical alteration and appears to reduce the activation energy of low-sulfur oil thermal chemical alteration by approximately 92 kJ mol^−1^^64^. Therefore, the observed acceleration of diamantanes generation is possibly due to sulfur-derived radical species or H_2_S formed via TSR or disproportionation reaction that enhances the formation of diamantanes.

The mechanism for generating diamondoids during TSR may be through free radical reactions, a mechanism similar to their generation from high temperature cracking of alkanes during the experiment simulation^65,66^. Consequently, we considered that the sulfur-derived radical species or H_2_S during TSR have a facilitative effect on the cleavage of high molecular-mass fractions, resulting in the new generation of diamondoids from TSR experiments in the present study. Meanwhile, hydrogen exchange between water and organic matter also proceeds via sulfur-derived radical species (i.e., from H_2_S)^53^, leading to demethylation and isomerization of hydrocarbon to form diamondoids. Briefly, TSR can lead to the generation of diamondoids through free radical reactions.

Notably, TSR resulted in the new generation of diamondoids (Fig. 5), and thus had a significant effect on the distribution and concentration of diamondoids. Thus, in TSR-altered oils, diamondoid-related maturity proxies have been altered significantly (Table 3), and thus cannot be used to indicate EOC.

#### Generation of thiadiamondoids during TSR

**Figure 11:**
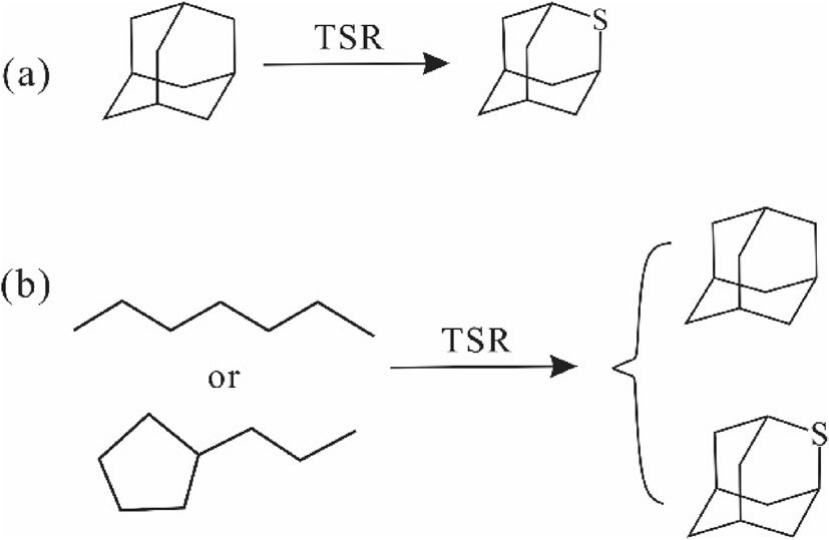
Possible pathways for the formation of diamondoids and thiadiamondoids from the TSR experiments. (**a**) Thiadiamondoids generated from diamondoids^37,38^; (b) thiadiamondoids generated from non-cage hydrocarbons.

**Figure 12:**
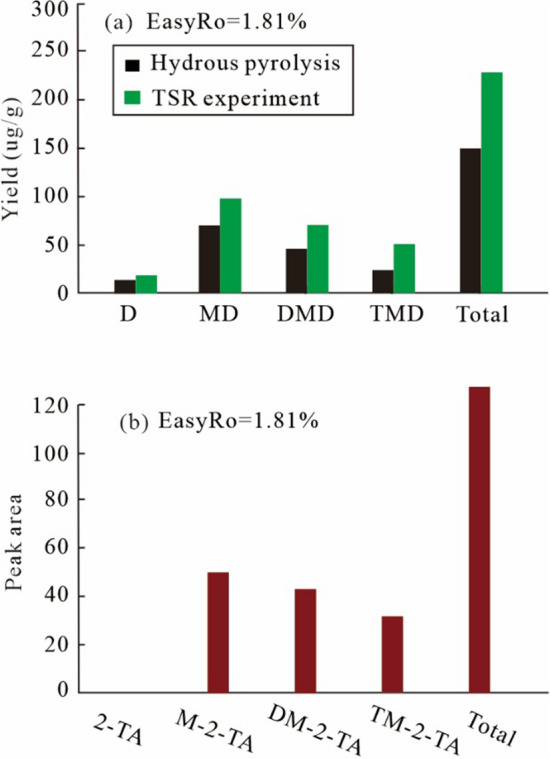
Different types of diamantanes and thiaadmantanes hydrocarbons at 1.81% EasyRo: (a) variation in the yields (µg/g oil) of different types of diamantanes from hydrous pyrolysis, anhydrous pyrolysis, and TSR experiments; (b) the relative concentration of thiaadmantnes from TSR experiments. hydrothermal pyrolysis, anhydrous pyrolysis, and TSR experiments (Group1); (b) the relative concentration of thiaadmantnes from TSR experiments. 2-TA = 2-thiaadmantane; M-2-TA = Methyl-2-thiaadmantane; DM-2-TA = Dimethyl-2-thiaadmantane; TM-2-TA = Trimethyl-2-thiaadmantane.Different types of diamantanes and thiaadmantanes hydrocarbons at 1.81% EasyRo: (a) variation in the yields (µg/g oil) of different types of diamantanes from hydrous pyrolysis, anhydrous pyrolysis, and TSR experiments; (b) the relative concentration of thiaadmantnes from TSR experiments.

Thiaadmantane and methyl thiaadmantanes isomers were detected at 1.81% EasyRo when the yields of diamantanes reached a maximum value during the hydrothermal pyrolysis of ZS1-L oil under TSR condition (Fig. 1). To our knowledge, this is the first successful laboratory synthesis of thiaadmantanes from a petroleum sample via TSR. Although previous laboratory experiments have successfully synthesized thiaadmantanes, thiaadmantanes were only detected from reactions of reduced S or CaSO_4_ with pure diamondoids^37,38^. Based on these laboratory experiments, Wei et al.^37^ proposed that diamondoids appear to be the only precursors of thiaadmantanes during TSR (Fig. 11a). Our results indicate that diamondoids are formed earlier than thiaadmantanes, thus, thiaadmantanes may have been generated from reactions of diamondoids with sulfur species. However, diamondoids can be formed from alkanes and it is hard to break C–C bonds in cage structure of diamondoids. These facts indicate that thiadiamondoids and diamondoids may have been generated simultaneously, likely not via reactions with diamondoids based on the following aspects (Fig. 11). Firstly, during TSR experiments at EasyRo of 1.81%, both diamantanes and corresponding thiaamantanes were formed, and thiadamantanes show positive correlations with the corresponding diamantanes (2-TA vs D; M-2-TA vs MD; DM-2-TA vs DMD; TM-2-TA vs TMD) from (Fig. 12a,b) with a higher yield of diamantanes during TSR compared with hydrothermal pyrolysis(TCA; Fig. 12a). The experimental results indicate that diamondoids and thiadamantanes may have been formed simultaneously, which is consistent with case studies showing the positive relationships between diamondoids and thiadiamondoids concentrations from oils and condensates from the Tarim Basin and Gulf of Mexico Basin^25,36^. In contrast, if diamondoids are the only precursor of thiaadamantane^37^, conversion of significant amounts of diamondoids to thiaadmantanes may lead to a negative correlation between the yields of diamondoids and thiaadmantanes. Secondly, C–C bonds in the cage structure of diamondoids have been proposed to be hard to break up due to their thermal stability^30–32^, it is more energy-favorable to form thiaadamantanes from other non-diamondoids compounds. Thus, it is reasonable for thiaadamantanes to have been generated during the formation of diamondoids. Considering that diamondoids can be generated from pyrolysis of all four fractions^13–16^, a non-diamondoid source of thiaadamantanes is proposed here as shown in Fig. 11b.

However, thiaadmantanes were only detected at 1.81% EasyRo (480 °C) not at other TSR experiments at temperatures from 336 °C to 600 °C. It is possible for thiaadamantanes to have been formed in relatively high-temperature conditions and are expected to decompose at higher EasyRo. Xiao et al.^67^ proposed that thiaadamantanes show slight to moderate cracking at EasyRo of 1.81%. In contrast, Wei et al.^7^ proposed that diamantane is stable up to 550 °C in the laboratory, which is consistent with the stability of adamantane reported by Oya et al.^68^, thus are thiaadamantanes are far less thermally stable than diamondoids. Similarly, thiadiamondoids were found to be thermally degraded at temperatures > 180 °C in TSR-altered oils from the Smackover and Norphlet formations of the US Gulf of Mexico^36,69^. The temperature of 180 °C can correspond to the equivalent vitrinite reflectance values of about 1.9% based on the thermal history of the Norphlet Sandstone in Mobile Bay, northern Gulf of Mexico^70^. Our TSR experimental results are generally consistent with this field observation. Considering that thiadiamondoids can be decomposed, their occurrence at the experiment at 480 °C suggests that the condition may be favorable for thiaadmantanes to be generated without being significantly decomposed. More simulation experiments are needed to verify this proposal.

## Conclusions

Based on our experiments, we can conclude that:

1. Hydrothermal pyrolysis experiments indicate that water can enhance the yields of diamondoids. Diamondoids may have mainly generated in 0.48% ~ 2.1% EasyRo and decomposed at > 2.1% EasyRo. Especially, diamantanes show decomposition at > 3.0% EasyRo.
2. MDI, EAI, DMAI-1, DMDI-2 are shown to be reliable maturity proxies at maturity over ca.1.0% EasyRo, and TMAI-1, TMAI-2 and DMAI-2 can only be used to reflect the higher maturity at EasyRo > 2.0%.
3. The extents of oil cracking (EOC) calculated from Dahl’s (3- + 4-) MD method are higher than the actual values, especially for highly mature samples due to their new generation, but can be obtained using our correction formula (EOC2 = 1.2402 × EOC1-28.952) at EasyRo < 3.0%.
4. EAI, DMDI-2, As/Ds, MAs/MDs, DMAs/DMDs, and DMAs/MDs can serve as molecular proxies to estimate the extent of oil cracking at EasyRo mainly < 2.1%.
5. TSR is found to newly generate diamantanes at < 1.81% EasyRo followed by their decomposition, while the decomposition of diamantanes by TCA occurs at > 2.62% EasyRo, and thus any diamondoid-related proxy cannot be used to reflect maturity and EOC.
6. Thiaadamantanes were generated from an experiment of TSR by oil at 1.81% EasyRo for the first time, likely via pyrolysis of non-diamondoid structure hydrocarbons.

Our results provide crucial experimental evidence for understanding the evolution of diamondoids during thermal maturity and TSR under natural conditions.

## Supplementary Information

Supplementary Legends.

Supplementary Information 2.
