# Supplementary material for: Diamondoids and thiadiamondoids generated from hydrothermal pyrolysis of crude oil and TSR experiments
Source: Sci Rep. 2022 Jan 7;12:196. doi: 10.1038/s41598-021-04270-z (PMC8742100; doi:10.1038/s41598-021-04270-z)
Supplement: Supplementary file 1 — Supplementary Legends. [file 41598_2021_4270_MOESM1_ESM.docx]

**Supplementary Table S1.** The yields (µg/g oil) of individual diamondoid compounds are identified in Table 1 at each heating temperature of hydrothermal and anhydrous pyrolysis experiments
