# Supplementary material for: Diamondoids and thiadiamondoids generated from hydrothermal pyrolysis of crude oil and TSR experiments
Source: Sci Rep. 2022 Jan 7;12:196. doi: 10.1038/s41598-021-04270-z (PMC8742100; doi:10.1038/s41598-021-04270-z)
Supplement: Supplementary file 2 — Supplementary Information 2. [file 41598_2021_4270_MOESM2_ESM.docx]

**Supplementary Table S1.**

| **Compound** | **Heating temperature (20℃/h)** | | | | | | | | | | | | | | | | | | | |
| --- | --- | --- | --- | --- | --- | --- | --- | --- | --- | --- | --- | --- | --- | --- | --- | --- | --- | --- | --- | --- |
|  | **312℃** | **336℃** | | **360℃** | | **384℃** | **382℃** | **408℃** | | **432℃** | | **456℃** | | **480℃** | | **504℃** | | **528℃** | | **552℃** |
|  | **a** | **a** | **b** | **a** | **b** | **a** | **b** | **a** | **b** | **a** | **b** | **a** | **b** | **a** | **b** | **a** | **b** | **a** | **b** | **b** |
| A | nd | nd | nd | 5.00 | nd | 10.26 | nd | 12.74 | 0.47 | 16.87 | 1.72 | 21.72 | 4.68 | 26.88 | 14.50 | 28.76 | 15.90 | 8.61 | 2.04 | nd |
| 1-MA | 5.20 | 10.03 | 0.09 | 15.24 | 0.22 | 18.27 | 0.34 | 22.14 | 3.28 | 28.62 | 7.78 | 36.31 | 11.60 | 38.89 | 35.30 | 42.52 | 47.10 | 20.66 | 17.10 | 0.15 |
| 2-MA | 1.27 | 4.96 | 0.50 | 12.29 | 0.11 | 16.48 | 0.21 | 19.02 | 0.75 | 22.71 | 4.20 | 30.38 | 16.00 | 37.38 | 44.90 | 40.10 | 37.30 | 14.98 | 2.72 | nd |
| 1,3-DMA | 5.93 | 10.23 | 0.15 | 15.37 | 0.15 | 23.30 | 0.19 | 24.50 | 0.61 | 25.27 | 1.96 | 27.63 | 6.74 | 35.70 | 20.90 | 45.34 | 33.70 | 34.63 | 24.30 | 0.94 |
| 1,4-DMA(cis) | 5.21 | 7.74 | 0.02 | 10.49 | 0.10 | 15.65 | 0.32 | 16.85 | 1.53 | 17.35 | 5.24 | 21.33 | 11.50 | 25.90 | 31.90 | 30.65 | 34.90 | 16.44 | 6.27 | 0.10 |
| 1,4-DMA(trans) | 4.91 | 7.54 | 0.10 | 10.19 | 0.12 | 15.72 | 0.36 | 16.85 | 0.67 | 17.85 | 3.73 | 20.23 | 11.20 | 25.13 | 30.90 | 30.83 | 35.00 | 16.84 | 6.23 | 0.17 |
| 1,2-DMA | 5.14 | 7.59 | 0.75 | 10.60 | 1.01 | 15.70 | 1.38 | 17.22 | 3.66 | 18.12 | 8.76 | 22.91 | 12.20 | 26.70 | 31.10 | 29.79 | 30.50 | 14.55 | 3.07 | nd |
| 2,6-+2,4-DMA | 5.48 | 8.39 | 1.09 | 9.32 | 1.42 | 11.48 | 1.83 | 12.27 | 2.57 | 14.21 | 5.47 | 18.48 | 14.70 | 21.89 | 31.60 | 19.74 | 16.50 | 11.32 | 0.94 | nd |
| 1,3,5-TMA | 3.81 | 6.62 | 0.17 | 10.58 | 0.22 | 15.23 | 0.21 | 16.55 | 0.37 | 17.07 | 0.61 | 17.90 | 1.15 | 20.33 | 4.97 | 28.66 | 8.64 | 29.73 | 10.00 | 0.72 |
| 1,3,6-TMA | 2.66 | 5.97 | 0.35 | 10.73 | 0.32 | 17.86 | 0.42 | 19.67 | 0.59 | 20.74 | 2.23 | 23.43 | 5.53 | 26.68 | 15.10 | 35.62 | 19.80 | 25.90 | 7.71 | 0.30 |
| 1,3,4-TMA(cis) | 7.21 | 8.71 | 0.81 | 9.64 | 0.89 | 13.83 | 1.17 | 14.74 | 1.36 | 15.96 | 2.96 | 17.36 | 6.02 | 27.24 | 14.30 | 33.33 | 17.10 | 23.37 | 5.66 | 0.12 |
| 1,3,4-TMA(trans) | 15.56 | 15.45 | 1.03 | 15.49 | 1.14 | 16.77 | 1.35 | 17.76 | 2.13 | 18.51 | 5.52 | 21.39 | 7.12 | 26.18 | 14.20 | 33.32 | 17.10 | 23.49 | 5.78 | 0.08 |
| 1,2,3-TMA | 21.02 | 23.99 | 2.33 | 25.43 | 3.00 | 25.90 | 3.29 | 26.38 | 4.20 | 28.77 | 7.78 | 33.86 | 15.00 | 37.98 | 30.30 | 39.11 | 24.20 | 20.02 | 1.33 | 0.05 |
| 1,3,5,7-TeMA | nd | 2.70 | nd | 5.48 | nd | 7.98 | 0.00 | 7.79 | nd | 8.58 | 0.00 | 8.07 | 0.00 | 9.80 | 0.68 | 14.46 | 1.06 | 17.52 | 0.31 | 0.78 |
| 1,2,5,7-TeMA | 8.21 | 10.39 | 0.86 | 10.25 | 0.94 | 11.27 | 1.06 | 12.26 | 0.95 | 12.85 | 1.68 | 14.49 | 2.87 | 16.95 | 7.49 | 23.07 | 11.30 | 21.95 | 7.03 | 0.29 |
| 1,3,5,6-TeMA | 5.10 | 6.85 | 0.59 | 6.56 | 0.86 | 7.12 | 0.16 | 8.71 | 0.16 | 8.53 | 1.08 | 8.75 | 0.58 | 10.47 | 1.54 | 14.84 | 1.73 | 16.98 | 0.28 | nd |
| 1,2,3,5-TeMA | 7.46 | 9.85 | 2.22 | 9.45 | 2.34 | 10.74 | 2.74 | 11.82 | 2.75 | 12.63 | 5.12 | 14.03 | 6.60 | 15.51 | 11.90 | 18.09 | 7.81 | 16.62 | 1.16 | nd |
| 1-E-3,5,7-TMA | 6.69 | 8.97 | 3.07 | 8.44 | 3.40 | 11.23 | 3.85 | 11.96 | 3.48 | 12.28 | 6.37 | 14.12 | 7.18 | 15.66 | 13.50 | 16.61 | 11.90 | 8.84 | 0.91 | nd |
| 1-EA | 3.83 | 4.29 | 0.52 | 4.25 | 0.73 | 4.21 | 0.87 | 4.33 | 1.92 | 5.71 | 3.58 | 7.76 | 6.62 | 7.06 | 12.80 | 5.44 | 11.10 | nd | 0.52 | nd |
| 2-EA | 8.49 | 9.90 | 2.00 | 9.71 | 2.59 | 8.47 | 2.83 | 8.63 | 3.81 | 9.16 | 6.75 | 11.05 | 10.50 | 8.81 | 13.80 | 4.51 | 3.23 | 2.74 | 0.20 | nd |
| 1-E-3-MA | 9.67 | 11.15 | 1.05 | 11.21 | 1.25 | 9.92 | 1.72 | 10.72 | 6.18 | 11.33 | 12.10 | 15.08 | 6.19 | 16.37 | 14.20 | 18.89 | 14.80 | 10.41 | 2.94 | nd |
| 1-E-3,5-DMA | 4.59 | 5.20 | 0.17 | 5.09 | 0.09 | 4.64 | 0.13 | 4.88 | 0.84 | 5.41 | 0.61 | 6.17 | 0.65 | nd | 2.73 | 10.27 | 3.30 | 8.85 | 1.00 | 0.06 |
| D | nd | 7.61 | nd | 14.16 | nd | 17.47 | nd | 20.77 | nd | 22.42 | nd | 27.51 | 5.69 | 23.93 | 8.26 | 16.58 | 10.70 | 15.49 | 9.29 | 1.04 |
| 4-MD | 13.36 | 15.54 | 15.20 | 15.32 | 17.00 | 15.84 | 16.40 | 15.76 | 15.80 | 15.42 | 17.40 | 18.76 | 18.50 | 20.65 | 23.60 | 28.20 | 26.20 | 30.71 | 25.50 | 8.63 |
| 1-MD | nd | nd | nd | 5.25 | nd | 9.48 | nd | 12.81 | nd | 15.07 | nd | 20.38 | 0.00 | 16.71 | nd | 14.65 | 13.00 | 13.29 | 10.40 | 0.89 |
| 3-MD | 23.68 | 30.42 | 17.40 | 30.89 | 20.70 | 30.43 | 19.90 | 31.64 | 21.80 | 33.50 | 22.00 | 37.74 | 17.40 | 37.47 | 27.00 | 37.44 | 43.90 | 38.74 | 33.30 | 5.07 |
| 4,9-DMD | 5.46 | 5.35 | 4.46 | 5.33 | 4.19 | 6.81 | 4.09 | 6.82 | 4.30 | 7.23 | 3.76 | 7.89 | 4.08 | 9.63 | 5.31 | 11.07 | 6.48 | 11.44 | 7.87 | 3.90 |
| 1,4-+2,4-DMD | 3.88 | 4.05 | 1.51 | 4.28 | 2.19 | 5.13 | 2.39 | 6.36 | 2.33 | 7.11 | 1.41 | 5.34 | 2.04 | 8.81 | 3.29 | 9.31 | 5.11 | 10.06 | 5.88 | 0.73 |
| 4,8-DMD | 7.66 | 8.33 | 6.53 | 9.51 | 7.64 | 10.07 | 8.29 | 10.14 | 7.39 | 11.17 | 7.88 | 8.30 | 3.33 | 8.69 | 3.58 | 9.22 | 4.80 | 9.68 | 4.85 | 0.67 |
| 3,4-DMD | 11.86 | 14.50 | 11.60 | 15.09 | 12.50 | 12.14 | 12.90 | 12.57 | 13.20 | 13.90 | 13.30 | 13.84 | 11.90 | 20.56 | 17.80 | 25.14 | 25.20 | 26.92 | 23.20 | 5.57 |
| 1,4,9-TMD | 2.41 | 3.16 | 1.29 | 3.93 | 1.37 | 3.64 | 1.37 | 3.12 | 1.21 | 4.77 | 1.36 | 4.36 | 0.97 | 5.91 | 1.19 | 6.85 | 1.85 | 7.62 | 2.29 | 0.61 |
| 3,4,9-TMD | 4.42 | 5.13 | 0.67 | 5.69 | 0.63 | 6.94 | 0.55 | 6.75 | 0.72 | 7.72 | 0.92 | 9.81 | 0.57 | 12.08 | 0.76 | 17.09 | 0.69 | 18.12 | 0.54 | nd |
| As | 137.44 | 186.51 | 17.87 | 230.83 | 20.90 | 292.02 | 24.43 | 317.78 | 42.28 | 348.54 | 95.25 | 412.44 | 164.63 | 477.51 | 398.61 | 563.95 | 403.97 | 364.44 | 107.50 | 3.76 |
| Ds | 72.74 | 94.09 | 58.66 | 109.46 | 66.22 | 117.95 | 65.89 | 126.74 | 66.75 | 138.30 | 68.03 | 153.92 | 64.48 | 164.44 | 90.79 | 175.55 | 137.93 | 182.07 | 123.12 | 27.11 |
| Total | 210.18 | 280.60 | 76.53 | 340.29 | 87.12 | 409.97 | 90.32 | 444.52 | 109.03 | 486.84 | 163.28 | 566.36 | 229.11 | 641.96 | 489.40 | 739.49 | 541.90 | 546.52 | 230.62 | 30.87 |
| 3-+4-MD(Ai/Mo) | 37.04 | 45.96 | 32.60 | 46.21 | 37.70 | 46.27 | 36.30 | 47.40 | 37.60 | 48.93 | 39.40 | 56.49 | 35.90 | 58.12 | 50.60 | 65.63 | 70.10 | 69.45 | 58.80 | 13.70 |
| 3-+4-MD(Ai/Mi) | 53.08 | 65.65 |  | 67.70 |  | 72.05 |  | 80.39 |  | 104.74 |  | 247.24 |  | 549.49 |  | 487.32 |  | 662.10 |  |  |
| EasyRo（%） | 0.48 | 0.57 | 0.57 | 0.68 | 0.68 | 0.79 | 0.78 | 0.96 | 0.96 | 1.19 | 1.19 | 1.47 | 1.47 | 1.81 | 1.81 | 2.19 | 2.19 | 2.62 | 2.62 | 3.06 |
| M0（g） | 0.0494 | 0.0510 |  | 0.0508 |  | 0.0531 |  | 0.0482 |  | 0.0424 |  | 0.0420 |  | 0.0338 |  | 0.0220 |  | 0.0196 |  |  |
| Mi（g） | 0.0345 | 0.0357 |  | 0.0347 |  | 0.0341 |  | 0.0284 |  | 0.0198 |  | 0.0096 |  | 0.0036 |  | 0.0030 |  | 0.0021 |  |  |
| EOC1 | 41.59 | 52.78 |  | 54.21 |  | 56.97 |  | 61.44 |  | 70.40 |  | 87.46 |  | 94.36 |  | 93.64 |  | 95.32 |  |  |
| EOC2 | 30.22 | 30.00 |  | 31.74 |  | 35.78 |  | 41.04 |  | 53.29 |  | 77.15 |  | 89.42 |  | 86.53 |  | 89.51 |  |  |
| EOC1-EOC2 | 11.38 | 22.78 |  | 22.47 |  | 21.19 |  | 20.40 |  | 17.11 |  | 10.31 |  | 4.93 |  | 7.11 |  | 5.81 |  |  |
| **Compound** | **Heating temperature (2℃/h)** | | | | | | | | | | | | | | | | |  |  |  |
|  | **312℃** | **336℃** | | **360℃** | | **384℃** | | **408℃** | | **432℃** | | **456℃** | | **480℃** | **504℃** | **528℃** | |  |  |  |
|  | **a** | **a** | **b** | **a** | **b** | **a** | **b** | **a** | **b** | **a** | **b** | **a** | **b** | **a** | **a** | **a** | **b** |  |  |  |
| A | nd | 4.29 | nd | 8.99 | 0.09 | 16.28 | 0.31 | 21.28 | 2.32 | 23.45 | 9.80 | 27.98 | 20.00 | 7.83 | 2.30 | 0.77 | nd |  |  |  |
| 1-MA | 5.77 | 10.79 | 0.30 | 20.14 | 1.09 | 28.43 | 3.86 | 35.62 | 7.25 | 38.64 | 25.80 | 42.28 | 50.70 | 39.10 | 8.72 | 4.87 | nd |  |  |  |
| 2-MA | 7.48 | 12.86 | 0.17 | 18.05 | 0.33 | 23.28 | 1.85 | 27.04 | 8.69 | 34.22 | 35.80 | 37.28 | 57.10 | 12.20 | 8.50 | 6.66 | nd |  |  |  |
| 1,3-DMA | 8.76 | 10.46 | 0.27 | 16.18 | 0.37 | 24.66 | 0.59 | 27.28 | 3.71 | 32.76 | 15.30 | 36.24 | 31.70 | 41.20 | 25.36 | 15.05 | nd |  |  |  |
| 1,4-DMA(cis) | 2.71 | 5.09 | 0.19 | 10.72 | 0.39 | 16.70 | 2.31 | 19.59 | 7.38 | 23.30 | 26.50 | 26.66 | 44.00 | 20.70 | 9.68 | 5.99 | 0.02 |  |  |  |
| 1,4-DMA(trans) | 5.72 | 8.32 | 0.27 | 11.72 | 0.12 | 16.14 | 1.35 | 18.41 | 7.10 | 23.65 | 26.60 | 27.32 | 43.90 | 21.30 | 9.76 | 5.99 | 0.03 |  |  |  |
| 1,2-DMA | 5.97 | 9.27 | 1.12 | 15.69 | 2.21 | 18.55 | 7.82 | 20.36 | 10.00 | 23.44 | 28.70 | 26.90 | 41.00 | 13.60 | 8.74 | 4.99 | nd |  |  |  |
| 2,6-+2,4-DMA | 5.97 | 7.14 | 1.59 | 10.33 | 1.93 | 11.61 | 3.09 | 14.61 | 9.69 | 15.13 | 30.50 | 20.81 | 36.80 | 2.08 | 7.98 | 4.98 | nd |  |  |  |
| 1,3,5-TMA | 4.74 | 8.70 | 0.18 | 11.63 | 0.26 | 15.78 | 0.32 | 16.74 | 0.43 | 18.92 | 3.63 | 20.20 | 8.05 | 13.30 | 26.24 | 20.03 | 0.12 |  |  |  |
| 1,3,6-TMA | 5.02 | 7.42 | 0.56 | 12.31 | 0.57 | 19.84 | 1.30 | 21.15 | 3.54 | 24.12 | 12.60 | 28.43 | 21.40 | 18.60 | 16.89 | 10.64 | 0.04 |  |  |  |
| 1,3,4-TMA(cis) | 8.57 | 11.00 | 1.14 | 14.30 | 1.14 | 14.97 | 2.45 | 15.02 | 4.75 | 20.60 | 13.00 | 26.33 | 20.00 | 14.90 | 15.02 | 10.61 | nd |  |  |  |
| 1,3,4-TMA(trans) | 13.72 | 14.58 | 1.25 | 16.87 | 1.51 | 16.93 | 3.87 | 19.30 | 6.40 | 22.12 | 14.10 | 25.90 | 19.30 | 14.40 | 14.94 | 10.59 | nd |  |  |  |
| 1,2,3-TMA | 19.82 | 19.69 | 3.37 | 25.34 | 3.85 | 28.33 | 6.13 | 32.88 | 11.50 | 34.90 | 31.10 | 38.00 | 40.00 | 7.88 | 12.83 | 10.59 | nd |  |  |  |
| 1,3,5,7-TeMA | nd | 3.03 | nd | 5.34 | nd | 6.88 | nd | 7.00 | nd | 8.17 | 0.45 | 10.69 | 0.93 | 1.70 | 14.92 | 10.99 | 0.14 |  |  |  |
| 1,2,5,7-TeMA | 9.98 | 10.49 | 1.12 | 11.67 | 1.09 | 11.69 | 1.81 | 12.85 | 2.70 | 12.50 | 6.94 | 16.32 | 11.10 | 12.70 | 16.66 | 10.67 | 0.14 |  |  |  |
| 1,3,5,6-TeMA | 6.68 | 7.10 | 0.06 | 7.38 | 0.11 | 7.01 | 0.83 | 7.47 | 0.83 | 8.43 | 1.42 | 9.30 | 2.31 | 1.96 | 12.53 | 10.59 | nd |  |  |  |
| 1,2,3,5-TeMA | 9.38 | 9.74 | 2.68 | 10.94 | 2.96 | 10.98 | 4.18 | 12.08 | 5.65 | 12.30 | 12.20 | 13.62 | 13.80 | 2.59 | 12.16 | 10.59 | nd |  |  |  |
| 1-E-3,5,7-TMA | 8.16 | 9.20 | 4.01 | 11.25 | 4.30 | 10.76 | 5.63 | 12.26 | 7.89 | 13.84 | 13.30 | 15.47 | 17.30 | 5.69 | 5.63 | 4.41 | nd |  |  |  |
| 1-EA | 3.14 | 2.91 | 0.94 | 3.79 | 1.03 | 3.86 | 2.77 | 5.93 | 5.26 | 6.02 | 11.80 | 6.41 | 15.60 | 3.79 | nd | nd | nd |  |  |  |
| 2-EA | 6.62 | 6.65 | 2.76 | 8.11 | 3.16 | 8.09 | 3.93 | 9.78 | 9.35 | 10.46 | 17.30 | 15.79 | 11.60 | 0.49 | 1.96 | 1.75 | nd |  |  |  |
| 1-E-3-MA | 7.88 | 8.49 | nd | 9.42 | nd | 10.01 | 1.69 | 13.50 | 4.35 | 14.43 | 12.80 | 16.03 | 18.20 | 9.80 | 5.92 | 4.40 | nd |  |  |  |
| 1-E-3,5-DMA | 4.24 | 4.46 | 0.11 | 4.99 | 0.07 | 4.87 | 0.19 | 5.67 | 0.83 | 6.69 | 2.47 | 7.45 | 4.09 | 2.64 | 6.11 | 4.39 | nd |  |  |  |
| D | nd | 8.65 | nd | 29.67 | nd | 30.60 | nd | 31.22 | nd | 25.30 | 7.90 | 18.78 | 12.20 | 15.00 | 15.82 | 5.93 | 0.20 |  |  |  |
| 4-MD | 11.50 | 11.57 | 19.70 | 13.86 | 17.30 | 15.57 | 20.30 | 17.26 | 23.80 | 25.79 | 23.10 | 29.16 | 27.10 | 34.60 | 36.54 | 27.34 | 0.65 |  |  |  |
| 1-MD | nd | 4.97 | nd | 8.79 | nd | 14.95 | nd | 20.70 | nd | 13.40 | nd | 15.55 | 13.40 | 16.10 | 15.46 | 8.64 | 0.26 |  |  |  |
| 3-MD | 20.87 | 22.12 | 25.80 | 27.82 | 25.90 | 29.33 | 25.30 | 35.42 | 23.40 | 35.48 | 26.80 | 36.76 | 45.30 | 53.40 | 37.75 | 24.19 | 0.99 |  |  |  |
| 4,9-DMD | 6.46 | 6.51 | 5.47 | 7.23 | 5.14 | 6.84 | 4.60 | 7.67 | 5.35 | 9.12 | 5.45 | 13.08 | 7.24 | 9.45 | 17.03 | 7.23 | 0.26 |  |  |  |
| 1,4-+2,4-DMD | 5.20 | 3.73 | 2.85 | 5.87 | 4.25 | 5.10 | 2.03 | 5.48 | 1.35 | 6.81 | 2.82 | 8.35 | 5.59 | 8.28 | 12.49 | 4.92 | 0.42 |  |  |  |
| 4,8-DMD | 9.23 | 6.64 | 10.20 | 9.54 | 11.00 | 8.67 | 9.33 | 8.82 | 11.60 | 9.59 | 3.72 | 10.84 | 5.43 | 7.33 | 11.68 | 4.79 | 0.27 |  |  |  |
| 3,4-DMD | 10.94 | 10.17 | 13.00 | 13.45 | 13.60 | 11.90 | 15.10 | 15.49 | 16.40 | 21.72 | 17.50 | 24.75 | 26.00 | 33.00 | 30.81 | 7.66 | 0.10 |  |  |  |
| 1,4,9-TMD | 3.34 | 3.64 | 1.53 | 4.21 | 1.67 | 3.72 | 1.69 | 3.82 | 1.38 | 5.71 | 1.68 | 7.51 | 1.71 | 2.70 | 8.71 | 4.84 | 0.55 |  |  |  |
| 3,4,9-TMD | 7.98 | 7.92 | 0.92 | 7.73 | 0.59 | 8.45 | 0.70 | 11.10 | 0.66 | 13.27 | 0.57 | 16.65 | 0.72 | 1.02 | 19.88 | 5.47 | nd |  |  |  |
| As | 150.35 | 191.69 | 22.09 | 265.17 | 26.58 | 325.65 | 56.28 | 375.82 | 119.62 | 428.09 | 352.11 | 495.40 | 528.88 | 268.45 | 242.86 | 169.56 | 0.49 |  |  |  |
| Ds | 75.51 | 85.91 | 79.47 | 128.19 | 79.45 | 135.13 | 79.05 | 156.98 | 83.94 | 166.20 | 89.54 | 181.42 | 144.69 | 180.88 | 206.16 | 101.01 | 3.70 |  |  |  |
| Total | 225.86 | 277.61 | 101.56 | 393.35 | 106.03 | 460.78 | 135.33 | 532.80 | 203.56 | 594.29 | 441.65 | 676.83 | 673.57 | 449.33 | 449.02 | 270.57 | 4.19 |  |  |  |
| 3-+4-MD(Ai/Mo) | 32.37 | 33.69 | 45.50 | 41.69 | 43.20 | 44.90 | 45.60 | 52.68 | 47.20 | 61.27 | 49.90 | 65.92 | 72.40 | 88.00 | 74.29 | 51.53 | 1.64 |  |  |  |
| 3-+4-MD(Ai/Mi) | 48.82 | 55.71 |  | 86.65 |  | 135.19 |  | 396.98 |  | 693.12 |  | 568.84 |  |  | 1670.83 | 815.25 |  |  |  |  |
| EasyRo（%） | 0.61 | 0.73 | 0.73 | 0.86 | 0.86 | 1.08 | 1.08 | 1.36 | 1.36 | 1.69 | 1.69 | 2.09 | 2.09 | 2.52 | 2.99 | 3.46 | 3.46 |  |  |  |
| M0（g） | 0.0504 | 0.0500 |  | 0.0495 |  | 0.0516 |  | 0.0505 |  | 0.0416 |  | 0.0413 |  |  | 0.0241 | 0.0238 |  |  |  |  |
| Mi（g） | 0.0334 | 0.0302 |  | 0.0238 |  | 0.0171 |  | 0.0067 |  | 0.0037 |  | 0.0048 |  |  | 0.0011 | 0.0015 |  |  |  |  |
| EOC1 |  | 44.35 |  | 64.22 |  | 77.07 |  | 92.19 |  | 95.53 |  | 94.55 |  |  | 98.14 | 96.20 |  |  |  |  |
| EOC2 |  | 39.53 |  | 51.89 |  | 66.79 |  | 86.73 |  | 91.16 |  | 88.41 |  |  | 95.55 | 93.68 |  |  |  |  |
| EOC1-EOC2 |  | 4.83 |  | 12.33 |  | 10.28 |  | 5.46 |  | 4.37 |  | 6.14 |  |  | 2.59 | 2.52 |  |  |  |  |

a unit: hydrous pyrolysis experiments.

b unit: hydrous pyrolysis experiments from Fang et al., (2012).

3-+4-MD(A_i_/M_o_): the yield of 3-+4-MD.

3-+4-MD(A_i_/M_i_): the concentration of 3-+4-MD.

EOC1: the calculated EOC (%) from (1- C_0_/C_c_) × 100.

EOC2: the actual EOC (%).
